# Prevalence of Pathogenic and Potentially Pathogenic Inborn Error of Immunity Associated Variants in Children with Severe Sepsis

**DOI:** 10.1007/s10875-021-01183-4

**Published:** 2022-01-01

**Authors:** Kate F. Kernan, Lina Ghaloul-Gonzalez, Jerry Vockley, Janette Lamb, Deborah Hollingshead, Uma Chandran, Rahil Sethi, Hyun-Jung Park, Robert A. Berg, David Wessel, Murray M. Pollack, Kathleen L. Meert, Mark W. Hall, Christopher J. L. Newth, John C. Lin, Allan Doctor, Tom Shanley, Tim Cornell, Rick E. Harrison, Athena F. Zuppa, Russel Banks, Ron W. Reeder, Richard Holubkov, Daniel A. Notterman, J. Michael Dean, Joseph A. Carcillo

**Affiliations:** 1 Division of Pediatric Critical Care Medicine, Department of Critical Care Medicine, Center for Critical Care Nephrology and Clinical Research Investigation and Systems Modeling of Acute Illness Center, Children’s Hospital of Pittsburgh, University of Pittsburgh, Pittsburgh, PA USA; 2 Division of Genetic and Genomic Medicine, Department of Pediatrics, Children’s Hospital of Pittsburgh, University of Pittsburgh, Pittsburgh, PA USA; 3 Genomics Core Laboratory, University of Pittsburgh, Pittsburgh, PA USA; 4 Department of Biomedical Informatics, University of Pittsburgh, Pittsburgh, PA USA; 5 Department of Genetics, Graduate School of Public Health, University of Pittsburgh, Pittsburgh, PA USA; 6 Department of Anesthesiology and Critical Care Medicine, Children’s Hospital of Philadelphia, Philadelphia, PA USA; 7 Division of Critical Care Medicine, Department of Pediatrics, Children’s National Hospital, Washington, DC USA; 8 Division of Critical Care Medicine, Department of Pediatrics, Children’s Hospital of Michigan, Detroit, MI USA; 9 Central Michigan University, Mt. Pleasant, MI USA; 10 Division of Critical Care Medicine, Department of Pediatrics, The Research Institute at Nationwide Children’s Hospital Immune Surveillance Laboratory, and Nationwide Children’s Hospital, Columbus, OH USA; 11 Division of Pediatric Critical Care Medicine, Department of Anesthesiology and Pediatrics, Children’s Hospital Los Angeles, Los Angeles, CA USA; 12 Division of Critical Care Medicine, Department of Pediatrics, St. Louis Children’s Hospital, St. Louis, MO USA; 13 Division of Pediatric Critical Care Medicine, The Center for Blood Oxygen Transport and Hemostasis, University of Maryland School of Medicine, MD Baltimore, USA; 14 Division of Critical Care Medicine, Department of Pediatrics, C. S. Mott Children’s Hospital, Ann Arbor, MI USA; 15 Department of Pediatrics, Lucile Packard Children’s Hospital Stanford, Stanford University, CA Palo Alto, USA; 16 Division of Critical Care Medicine, Department of Pediatrics, Mattel Children’s Hospital at University of California Los Angeles, Los Angeles, CA USA; 17 Department of Pediatrics, University of Utah, Salt Lake City, UT USA; 18 Department of Molecular Biology, Princeton University, Princeton, NJ USA

**Keywords:** Sepsis, Inborn errors of immunity, Hyperinflammation, Primary immunodeficiency

## Abstract

**Purpose:**

Our understanding of inborn errors of immunity is increasing; however, their contribution to pediatric sepsis is unknown.

**Methods:**

We used whole-exome sequencing (WES) to characterize variants in genes related to monogenic immunologic disorders in 330 children admitted to intensive care for severe sepsis. We defined candidate variants as rare variants classified as pathogenic or potentially pathogenic in QIAGEN’s Human Gene Mutation Database or novel null variants in a disease-consistent inheritance pattern. We investigated variant correlation with infection and inflammatory phenotype.

**Results:**

More than one in two children overall and three of four African American children had immunodeficiency-associated variants. Children with variants had increased odds of isolating a blood or urinary pathogen (blood: OR 2.82, 95% CI: 1.12–7.10, *p* = 0.023, urine: OR: 8.23, 95% CI: 1.06–64.11, *p* = 0.016) and demonstrating increased inflammation with hyperferritinemia (ferritin $\ge 500$ ng/mL, OR: 2.16, 95% CI: 1.28–3.66, *p* = 0.004), lymphopenia (lymphocyte count < 1000/µL, OR: 1.66, 95% CI: 1.06 – 2.60, *p* = 0.027), thrombocytopenia (platelet count < 150,000/µL, OR: 1.76, 95% CI: 1.12–2.76, *p* = 0.013), and CRP greater than 10 mg/dl (OR: 1.71, 95% CI: 1.10–2.68, *p* = 0.017). They also had increased odds of requiring extracorporeal membrane oxygenation (ECMO, OR: 4.19, 95% CI: 1.21–14.5, *p* = 0.019).

**Conclusion:**

Herein, we describe the genetic findings in this severe pediatric sepsis cohort and their microbiologic and immunologic significance, providing evidence for the phenotypic effect of these variants and rationale for screening children with life-threatening infections for potential inborn errors of immunity.

**Supplementary Information:**

The online version contains supplementary material available at 10.1007/s10875-021-01183-4.

## Introduction

Severe sepsis remains a leading cause of morbidity and mortality worldwide with 40 million annual cases, contributing to 60% of pediatric deaths, emphasizing a need for pathobiological insight [1]. Inborn errors of immunity (IEI) are hypothesized to underlie vulnerability to life-threatening infection, not just in primary immunodeficiencies, but also in sporadic cases of severe sepsis [2]. While links have been explored in individual cases and pathogens such as influenza [3], invasive pneumococcus [4], *Pseudomonas* [5], SARS-CoV-2 [6, 7], and previously healthy children with bacteremia [8], systematic investigation of IEI in pediatric sepsis is limited.

Next-generation sequencing (NGS) advances have expanded our understanding of the molecular basis of IEI. Currently, the International Union of Immunological Societies (IUIS) updates its catalog of monogenic immunological disorders biannually and describes over 400 genetic defects [9]. Due to phenotypic and genetic heterogeneity, WES is commonly used in their diagnosis. However, the broader application of WES to life-threatening infection has been hindered by challenges in variant interpretation. Additionally, even pathogenic variants are impacted by penetrance and expressivity and may not cause disease in all individuals [10]. This leads to a critical knowledge gap in understanding the prevalence and impact of IEI in severe pediatric sepsis.

In neonates, a genetic disease contributes significantly to morbidity and mortality, and NGS has demonstrated measurable clinical impact [11, 12]. In older children with concerns for genetic disease, diagnostic rates approach 40% and can be achieved in less than 2 weeks in research settings. The two largest NGS series in pediatric intensive care unit (PICU) patients both included cases of immunodeficiency with life-threatening infections [13, 14].

Building on these studies, we used WES to test the hypothesis that variants in genes in the 2019 IUIS Primary Immunodeficiency classification are common among children with severe sepsis and associate with infectious and inflammatory phenotypes.

## Methods

### Subject Selection

Children with severe sepsis between 44 weeks and 18 years old admitted to one of nine PICUs in the Eunice Kennedy Shriver National Institute of Child Health and Human Development Collaborative Pediatric Critical Care Research Network (NICHD-CPCCRN) between 2015 and 2017 without advance directives limiting care were eligible. The study was approved by the central and all nine local institutional review boards. Written informed consent was obtained from one or more parents/guardians, and child assent was garnered when able. At each center, all PICU admissions were screened twice weekly for the presence of sepsis, $\ge$ 1 organ failure and a central venous or arterial catheter. Study enrollment occurred a median of 2 days after ICU admission (IQR: + 1 to + 4 days after ICU admission). Per-site enrollment was limited to 80 subjects. Sepsis was defined as suspected or documented infection and two systemic inflammatory response criteria: (1) tachycardia (heart rate > 90th percentile for age); (2) tachypnea (respiratory rate > 90th percentile for age); (3) temperature (< 36 °C or > 38.5 °C); and (4) abnormal WBC count (> 12,000/µL or < 4000/µL or > 10% immature neutrophils). Severe sepsis was defined by the previously mentioned criteria and at least one organ failure (cardiovascular: need for cardiovascular infusion support; pulmonary: need for mechanical ventilation support with PaO2/FIO2 ratio < 300 without support; hepatic: total bilirubin > 1.0 mg/dl and alanine aminotransferase > 100 IU/L; renal: serum creatinine > 1.0 mg/dl and oliguria (urine output < 0.5 mL/kg/h); hematologic: thrombocytopenia < 100,000/uL and international normalized ratio > 1.5 × normal; and CNS: Glasgow Coma Scale < 12 without sedatives) [15]. No geneticists or intensivists were involved in participant selection and suspicion for immunologic disorders was not considered at enrollment. Sequencing was performed between 2018 and 2020 and was not available to treating physicians.

### DNA Extraction and Exome Sequencing

**Fig. 1:**
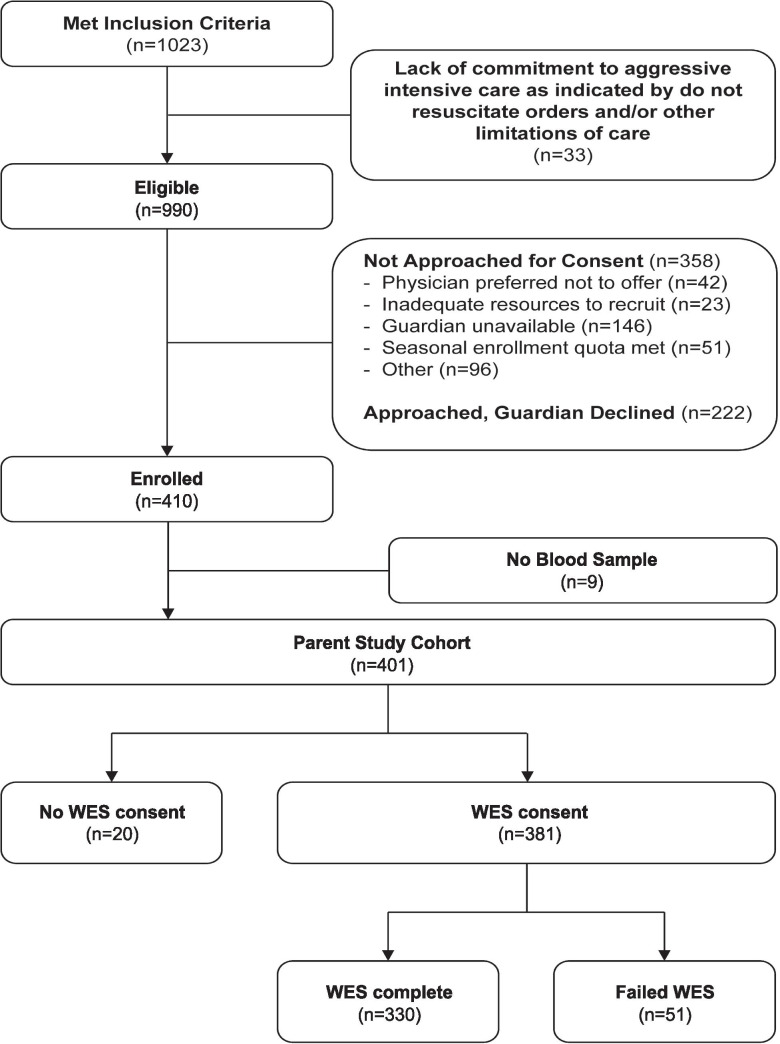
Consort diagram for the study. Diagram of recruitment, inclusion, and exclusion criteria

A total of 401 pediatric patients were enrolled, for whom 381 parents (95%) provided WES consent. 2 mL of whole blood were collected for DNA extraction using standard methods. A total of 330 children completed WES ($\mathrm{median}\;\mathrm{DNA}\;\mathrm{yield}\;39.24\mu\mathrm g,\;\mathrm{IQR}:\;20.19\mu\mathrm g-71.49\mu\mathrm g$) and 51 had insufficient DNA ($\mathrm{median}\;\mathrm{yield}\;1.120\mu\mathrm g,\;\mathrm{IQR}:\;0.205\mu\mathrm g-3.455\mu\mathrm g,\;p<0.0001$; Fig. 1). Those with insufficient DNA extraction had a lower median lymphocyte count on the day of sequencing blood draw (230 vs. 1200 cells/μl, *p* = 1.43 × 10^−10^), were older (median 8.5 y vs. 5.3 y, *p* = 0.0008), less likely to be previously healthy (13.7% vs. 47.9%, *p* = 2.8 × 10^−6^), more likely to have malignancy (47.1% vs. 7.0%, *p* = 9.21 × 10^−12^), and suffered higher mortality (23.5% vs. 8.5%, *p* = 0.0028).

The University of Pittsburgh Genomics Research Core performed WES on the Ion Torrent platform. Libraries were constructed using the Ampliseq Exome RDY (Thermo Fisher Scientific) with 100 × target coverage. FASTQ files were aligned to *Homo sapiens* reference sequence GRCh37/hg19 to generate VCF files. Fabric Genomics’ Opal software (Fabric Genomics Inc., California) was used to identify nonsynonymous variants including missense, nonsense, frameshift, start, or splice site (+ / − 2 bp) mutations. Candidate variants were filtered for a minimum coverage > 10 × and a PHRED score > 20 for quality control. No parental DNA samples were collected. Sanger sequencing proved prohibitive due to variant prevalence and was therefore not performed.

### Candidate Gene Filter

The IUIS IEI report currently lists 430 genes as causes of 403 monogenic primary immunodeficiencies [9] categorized in nine groups: complement disorders, autoinflammatory disorders, combined immunodeficiencies with associated or syndromic defects, congenital defects of phagocyte number and function, disorders of immune dysregulation, defects in innate and intrinsic immunity, marrow failure, predominantly antibody deficiencies, and immunodeficiencies affecting cellular and humoral immunity (Table S1). Candidate variants were restricted to this list, with minor allele frequency (MAF) < 0.05 in ExAC, 1000 Genome, NHLBI-ESP 6500, and gnomAD databases. Variants were required to exhibit a disease-consistent inheritance pattern (one variant for autosomal dominant or *X*-linked in males, and two variants for autosomal recessive). We required only a single heterozygous variant for disorders with evidence supporting both recessive and dominant inheritance. Additionally, we limited missense variants to those classified as disease mutation (DM) or disease mutation? (DM?) in QIAGEN’s Professional Human Gene Mutation Database (HGMD) based on peer-reviewed literature of the variant in human disease. The DM? designation indicates a potential but not definitive association and is an uncertain link between variant and disease. In this manuscript, DM and DM? variants are defined as pathogenic and potentially pathogenic, respectively. As our dataset is without functional validation, we utilized HGMD for annotation where evidence for variant classification can be reviewed. We also aimed to increase power to detect genotype–phenotype associations in line with this resource’s aim to minimize false negatives [16]. While HGMD professional is commonly cited as evidence for variant pathogenicity, it is not equivalent to clinical sequencing which integrates additional annotation resources including ClinVar and others. Therefore, while the amino acid changes in this manuscript are reported in peer-reviewed literature, they are not equated to immunodeficiency diagnosis. Complete references for individual variants are listed in Table S2. Unique null (nonsense, frameshift, + / − 1 or 2 splice site and initiation codon) variants found in a disease-causing inheritance pattern were treated as pathogenic per the American College of Medical Genetics (ACMG) standards and guidelines for variant interpretation [17]. Highly recurrent null variants were filtered from the dataset, due to the high likelihood that they represented sequencing error or had no impact on function.

### Association Testing with Microbiologic and Inflammatory Markers

We tested for association between any IEI variant or IUIS group and infection site and inflammatory markers. All microbiologic/virologic data were limited to 48 h before or after enrollment. All results were reviewed, and likely contaminants were omitted by site including coagulase-negative staphylococci and viridans group streptococci blood cultures, *Candida albicans* and viridans group streptococci respiratory cultures, and mixed flora urine cultures. Culture-negative sepsis was defined as the absence of positive viral or bacterial testing from any site during the 48 h before or after enrollment after contaminant exclusion. For markers of inflammation, the most abnormal value per subject was used to define lymphopenia (minimum lymphocyte count < 1000/µL), hyperferritinemia (ferritin > 500 ng/mL), both markers of inflammation and risk for sepsis mortality [18, 19], thrombocytopenia [20] (platelet count < 150,000/µL) and extreme elevations in C-reactive protein (CRP > 10 mg/dL, normal 0.04–0.79 mg/dL) [21].

### Statistical Methods

Comparisons between baseline characteristics and outcome were made between children with and without IEI using $\chi^2$ testing for categorical variables and Wilcoxon rank sum for continuous variables. For IUIS group comparisons to children without identified variants, Fisher exact testing was performed. For IUIS group comparisons, both unadjusted and adjusted *p*-values following multiple testing correction for the number of groups are reported (Benjamini–Hochberg method).

For individual identified variants, a cohort minor allele frequency (MAF) was computed and compared using $\chi^2$ testing to gnomAD (https://gnomad.broadinstitute.org/v2.1.1), a sequencing database of 141,456 individuals that excludes those with severe pediatric diseases [22]. This approach has been used to identify overrepresented variants in rare disease cohorts [23]. Additional MAF comparisons were made based on the self-reported race of any ethnicity between (1) African American and non-African American children in the sepsis cohort and (2) African American children in the sepsis cohort and African participants in gnomAD. *p*-values were multiple-test corrected using the Benjamini–Hochberg method for the number of identified variants.

All statistics were completed in R version 4.0.4 using a *p-*value threshold of < 0.05.

## Results

### Prevalence of Inborn Error of Immunity Associated Pathogenic and Potentially Pathogenic Variants in Children with Severe Sepsis

**Fig. 2:**
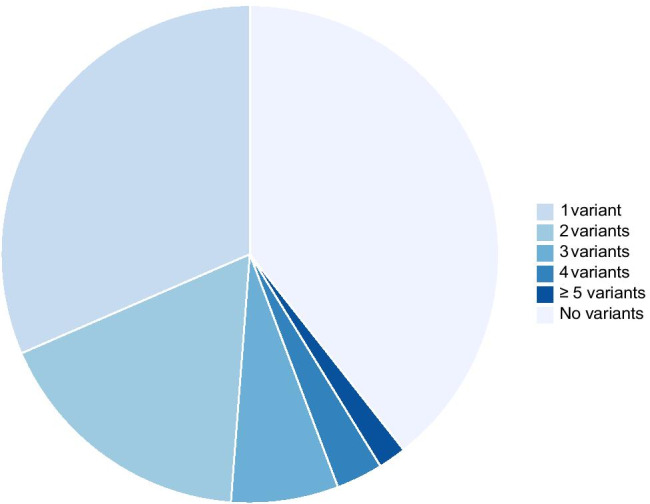
Distribution of the number of variants per subject in the severe pediatric sepsis cohort. Shows the number of inborn errors of immunity variants identified per subject, which ranged from zero to eight

Among the 330 sequenced, variants occurred in 200 children (61%) including 89 previously healthy children (Table 1). More than 3/4 of African American children with severe sepsis were found to have a genetic variant previously associated with IEI (*p* = 0.004). While most individuals harbored single IEI (*N* = 104, 52% of positive WES), 57 children (29%) had two variants, 23 had three (12%), and 16 had five or more (8%, Fig. 2, Table S3). Additionally, children with IEI had increased odds of requiring extracorporeal membrane oxygenation (ECMO, OR 4.19, 95% CI: 1.21–14.50, *p* = 0.019). Other interventions including mechanical ventilation, continuous renal replacement therapy, and plasma exchange did not differ between groups.

**Table 1:** Characteristics for all study participants, individuals with and without inborn errors of immunity on the whole-exome sequencing

	**All (*N* = 330)**	**IEI WES positive (*N* = 200)**	**IEI WES negative (*N* = 130)**	***p*-value**
Patient characteristics				
Age (years)	5.3 (1.3–11.9)	5.9 (1.5–12.3)	4.9 (0.9–10.5)	NS
Male	179 (54.2%)	113 (56.5%)	66 (50.7%)	NS
Race				
White	224 (67.9%)	128 (64.0%)	96 (73.8%)	NS
Black	70 (21.2%)	53 (26.5%)	17 (13.1%)	0.004^a^
Other	17 (5.2%)	11 (5.5%)	6 (4.6%)	NS
Not reported	22 (6.7%)	10 (5.0%)	12 (9.2%)	NS
Ethnicity				
Hispanic or Latino	51 (15.5%)	25 (12.5%)	26 (20%)	NS
Not Hispanic or Latino	266 (80.6%)	166 (83.0%)	100 (77.7%)	NS
Unknown	13 (3.9%)	9 (4.5%)	4 (3.1%)	NS
Previously healthy	158 (47.9%)	89 (44.5%)	69 (53.0%)	NS
Malignancy	23 (7.0%)	17 (8.5%)	6 (4.6%)	NS
Patient outcomes				
ICU mortality	28 (8.5%)	18 (9.0%)	10 (7.7%)	NS
ICU LOS, days	12 (7–20.0)	12.0 (7.0–22.0)	11.5 (6.0–18.0)	NS
MV	302 (91.5%)	181 (90.5%)	121 (93.1%)	NS
Intubated	161 (48.8%)	99 (49.5%)	62 (47.6%)	NS
CRRT	34 (10.3%)	23 (11.5%)	11 (8.5%)	NS
ECMO	21 (6.4%)	18 (9.0%)	3 (2.3%)	0.019^b^
PLEX	18 (5.5%)	12 (6.0%)	6 (4.6%)	NS

Categorical data are expressed as *N* (%). Continuous variables are presented as median and IQR. Statistical comparison between groups was performed using Wilcoxon rank sum for continuous variables and $\chi$ ^2^ test for categorical variables. There were no significant differences between the complete cohort and IEI positive or negative groups. In comparing children with severe sepsis with IEI variants to those without, children of African American ancestry were noted to have increased odds of having an IEI variant identified. Children with IEI variants also had increased odds of requiring extracorporeal membrane oxygenation (ECMO)

a. OR 2.39 95% CI 1.31–4.36, *p* = 0.004

b. OR 4.19 CI 1.21–14.5, *p* = 0.019

*IEI*, inborn errors of immunity; *IQR*, interquartile range; *N*, number; *ICU*, intensive care unit; *LOS*, length of stay; *MV*, mechanical ventilation; *ECMO*, extracorporeal membrane oxygenation; *PLEX*, plasma exchange; *CRRT*, continuous renal replacement therapy

**Fig. 3:**
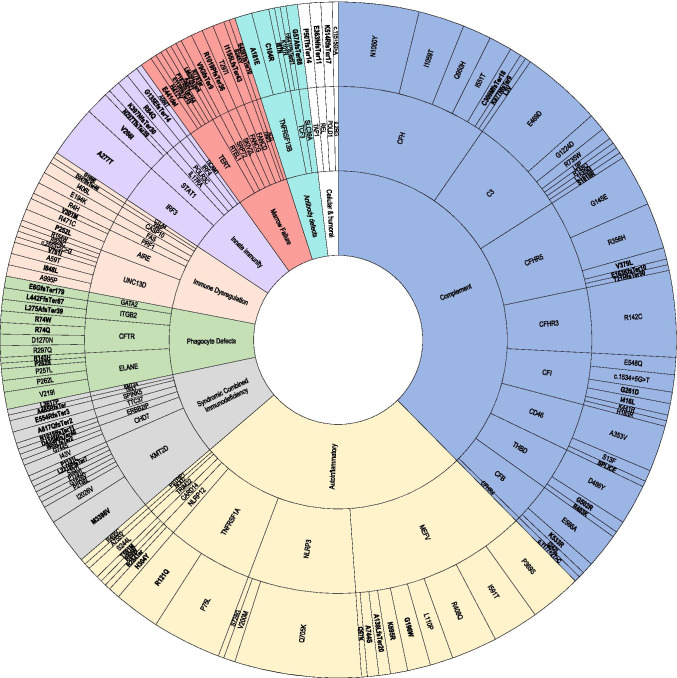
Genomic landscape of inborn errors of immunity in severe pediatric sepsis. This figure displays every pathogenic or potentially pathogenic variant identified in the cohort, and from the inner- to the outermost ring, it indicates the variants’ International Union of Immunologic Societies Classification, the disease where the variant has been previously identified, the gene locus affected, and finally, the specific allele change. A total of 131 unique variants were identified in 200 individuals. Representatives of all IUIS disease classes were identified in the cohort and included from the most to the least common: complement deficiencies (complement, blue), autoinflammatory disorders (autoinflammatory, yellow), combined immunodeficiency with associated or syndromic features (syndromic features, gray), congenital defects of phagocyte number and function (phagocyte defects, green), diseases of immune dysregulation (immune dysregulation, peach), defects of innate and intrinsic immunity (innate immunity, purple), bone marrow failure disorders (marrow failure, red), predominantly antibody deficiencies (antibody, teal), and immunodeficiencies affecting cellular and humoral immunity (T and B cell, white). Variants assigned DM in the HGMD database are shown in bold, while those with DM? designations are shown in plain font face. The wedge size allotted to an individual variant, gene, disease, and immune disorder classification is proportional to the relative frequency of its observation in the dataset

The variants exhibited both locus and allele diversity including 333 total variants at 131 loci (Fig. 3.). Among these loci, 69 were associated with autosomal dominant disorders (*N* = 144 individuals), 25 with autosomal recessive conditions (homozygous or compound heterozygous in *N* = 21 individuals), and two with *X*-linked recessive disorders (*N* = 2 individuals). A total of 35 loci were associated with disorders that can be inherited as either dominant or recessive conditions (*N* = 88 individuals).

### IEI Variants According to the International Union of Immunologic Society Functional Class

In this cohort, we identified diverse IEI variants across all nine IUIS categories. Complement variants implicated in atypical hemolytic uremic syndrome (aHUS) were the most common, identified in 92 individuals (Fig. 3). The most common variants included *CFH* (c.3148A > T; p.Asn1050Tyr), *C3* (c.1407G > C; p.Glu469Asp), and *CFHR3* (c.424 C > T; p.Arg142Cys), all previously described in aHUS [24, 25, 26, 27, 28]. We also identified other alternative complement variants reported in aHUS in *CFHR5, CFI, CD46, CFB, CFHR3, THBD,* and *CFHR4*.

The next most frequent variant group was related to autoinflammatory conditions (Fig. 3). They occurred in 77 individuals and included variants reported in familial Mediterranean fever (*MEFV*), familial cold inflammatory syndrome type 2 (*NLRP12*), TNF receptor-associated periodic syndrome (*TNFRSF1A*), Blau syndrome (*NOD2*), pyogenic sterile arthritis, pyoderma gangrenosum acne syndrome (*PSTPIP1*), and CARD14-mediated pustular psoriasis (*CARD14*). The most common variant in this group, *NLRP3* c.2113C > A; p.Gln705Lys was carried by 21 individuals and homozygous in one.

The remaining other pathogenic or potentially pathogenic variants according to IUIS Classification of Primary Immunodeficiencies include combined immunodeficiencies with associated or syndromic features (*KMT2D, CHD7, ERBB2IP, TTC37, SPINK5, TBX1, KMT2A*); congenital defects of phagocyte number and function (*ELANE, CFTR, ITGB2, GATA2*); disorders of immune dysregulation (*UNC13D, AIRE, PRF1, FAS, CASP10, XIAP, CTLA4*); defects of innate and intrinsic immunity (*IRF3, STAT1, IL17RA, POLR3C, IRF4, TICAM1*); bone marrow failure (*TERT, RTEL1, SRP72, SKIV2L, FANCG, FANCD, TINF2, ACD*); predominantly antibody deficiencies (*TNFRSF13B, TCF3, SLC39A)*; and mutations affecting cellular and humoral immunity (*TAP1, REL, POLD1, IL2RG*) as shown in Fig. 3. Variant data including zygosity, predicted in silico impact, and MAF can be found in Supplementary Table S3. Phenotypic details for novel variants are found in Table S4.

### IEI Status and IUIS Group Association with Infection Site and Laboratory Markers of Inflammation

**Fig. 4:**
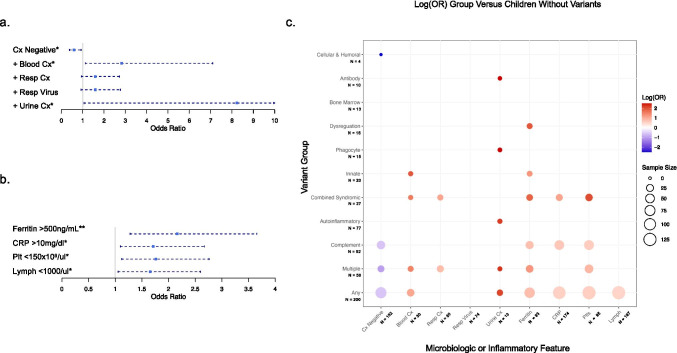
Infection types and immunologic characteristics for individuals with and without inborn errors of immunity. **a** Shows the odds ratios (OR) and 95% CI for microbiologic testing in children with any inborn error of immunity compared to children with no variants identified. Microbiologic data includes all viral testing and bacterial culture data + / − 2 days from enrollment with likely contaminants removed. Respiratory cultures include specimens taken from tracheal aspirate, pleural fluid culture, bronchial brush, and bronchoalveolar lavage. Children with variants were noted to have significantly increased odds of positive blood and urine culture, as well as decreased odds of culture-negative sepsis. b Shows the odds ratios and 95% CI for inflammatory laboratory characteristics in children with any inborn error of immunity compared to children with no variants identified. Comparison is of the most extreme values from any day on the study. Children with a potential inborn error of immunity variants were noted to have significantly increased odds of hyperferritinemia, elevated CRP, thrombocytopenia, and lymphopenia. c Shows the log(OR) for each microbiologic and inflammatory laboratory feature by IUIS variant group compared to children with no variant identified. Only ORs that differed significantly from children with no identified variants are shown (p < 0.05, unadjusted). Following adjustment for multiple test correction the following ORs remained significantly different from children with no variants identified: (1) any: hyperferritinemia; (2) multiple: culture negative, positive blood culture, hyperferritinemia; (3) complement: hyperferritinemia; (4) combined syndromic: hyperferritinemia; (5) innate: hyperferritinemia; (6) phagoctye: positive urine culture; (7) dysregulation: hyperferritinemia). For example, children with any variant, variants from multiple IUIS classes, combined syndromic and innate immune IUIS classes had increased odds of positive blood cultures compared to children with no variants identified. Similarly, children with any variant, variants from multiple classes, complement variants, and combined cellular and humoral defects had decreased odds of having culture-negative sepsis. Multiple: children with variants from more than one IUIS class, Cx: culture, *p < 0.05, **p < 0.01

IEI-variants associated with infection site (Fig. 4a). Children with variants had increased odds of positive blood or urinary culture (blood: OR 2.82, 95% CI 1.12–7.10, *p* = 0.022; urine: OR: 8.23, 95% CI 1.06–64.11, *p* = 0.016) and were less likely to have culture-negative sepsis (OR: 0.59, 95% CI: 0.38–0.92, *p* = 0.020). There was no significant difference in odds of positive respiratory culture or respiratory viral testing. As seen in Fig. 4b, children with IEI-associated variants were more likely to be lymphopenic (OR: 1.67, 95% CI: 1.05–2.60, *p* = 0.027), hyperferritinemic (OR: 2.16, CI: 1.28–3.67, *p* = 0.004), thrombocytopenic (OR: 1.76, 95% CI: 1.12–2.76, *p* = 0.013), and have a CRP > 10 mg/dl (OR: 1.71, 95% CI: 1.09–2.68, *p* = 0.017).

In comparison to children without identified variants, infection type and inflammatory markers also varied by the IUIS group (Fig. 4c). Individuals with variants from multiple IUIS classes had increased odds of positive blood, respiratory, or urine culture and increased odds of hyperferritinemia, lymphopenia, thrombocytopenia, and CRP > 10 mg/dl. Children with complement variants were less likely to have culture-negative sepsis and had increased odds of hyperferritinemia, thrombocytopenia, and CRP > 10 mg/dl. Individuals with autoinflammatory variants had increased odds of positive urine culture. Individuals with combined immunodeficiencies with syndromic features had increased odds of positive blood or respiratory cultures, hyperferritinemia, thrombocytopenia, and CRP > 10 mg/dl. Those innate immune disorder variants had increased odds of positive blood culture and hyperferritinemia, while those with phagocytic disorder variants had increased odds of positive urine culture. Individuals with immune dysregulation variants had the highest odds of hyperferritinemia seen in any group (OR 7.00: 95% CI: 2.3–21.1, *p* = 0.0005). Those with antibody deficiencies had increased odds of positive urine culture, and those with variants affecting cellular and humoral immunity had decreased odds of culture-negative sepsis. Following adjustments for multiple testing, the following comparisons remained significantly different from children with no variants: (1) any variant: hyperferritinemia; (2) multiple variants: culture negative, blood culture positive, and hyperferritinemia; (3) complement: hyperferritinemia; (4) combined syndromic: hyperferritinemia; (5) innate: hyperferritinemia; (6) phagoctye: positive urine culture; and (7) dysregulation: hyperferritinemia (Tables S5 and S6).

### Variants Overrepresented in the Pediatric Sepsis Cohort Compared to gnomAD

After candidate variant identification, we sought statistical evidence of variant overrepresentation in children with severe sepsis compared to gnomAD [22] (Table 2, Table S7). Three complement variants were significantly overrepresented: *C3* c.1407G > C; p.Glu469Asp (adjusted *p* = 1.2 × 10^−7^), *C3* c.443G > A; p.Arg148Gln (adjusted *p* = 4.5 × 10^−7^) both activating variants of the main complement convertase [27] and *CFHR3* c.424C > T; p.Arg142Cys (adjusted *p* = 2.6 × 10^−3^), a negative regulator of complement [29]. In addition, *IRF3* (c.829G > A; p.Ala277Thr), a transcriptional regulator of type I interferon (IFN)-dependent immune responses [30], was overrepresented in the sepsis cohort (adjusted *p* = 0.012).

**Table 2:** Summary table of overrepresented inborn errors of immunity alleles by ancestry groups following multiple test corrections

Variant	MAF ratio: sepsis/gnomAD, (*N*)	Adj *p*-value	MAF ratio: African American sepsis/non-African American sepsis, (*N*)	Adj *p*-value	MAF ratio: African American sepsis/ gnomAD African, (*N*)	Adj *p*-value
*C3* c.1407G > C p.Glu469Asp	4.7, (*N* = 14)	1.2 × 10^−7^	22.3, (*N* = 12)	4.4 × 10^−5^		
C3 c.443G > A p.Arg148Gln	214.2, (*N* = 1)	4.5 × 10^−7^			0.014/0, (*N* = 1)	4.08 × 10^−9^
CFH c.2850G > T p.Gln950His					18.1, (*N* = 2)	9.73 × 10^−6^
*CFHR3* c.424C > T p.Arg142Cys	2.9, (*N* = 15)	2.6 × 10^−3^	14.9, (*N* = 12)	2.3 × 10^−6^		
*CFHR5* c.434G > A p.Gly145Glu			29.7, (*N* = 8)	7.3 × 10^−5^		
*IRF3* c.829G > A p.Ala277Thr	3.4, (*N* = 9)	0.012				
*TNFRSF1A* c.224C > T p.Pro75Leu			18.6, (*N* = 10)	1.4 × 10^−5^		

Summary table of statistically significant frequency comparisons following multiple test corrections between groups: (1) sepsis cohort and gnomAD to identify variants more common in the sepsis population as a whole; (2) African American and non-African American children in the sepsis cohort to identify variants that may explain the increased odds of potential IEI variants identified in African American children; (3) African Americans in the sepsis cohort and individuals of African ancestry in gnomAD to identify overrepresented variants on a specific ancestral background. Table shows the ratio of minor allele frequency in statistically significant comparisons of interest and the number of individuals within the subgroup with the variant. *p*-values shown are *χ*^2^ comparisons with Benjamini–Hochberg adjustment for the total number of unique variants observed in the dataset (*N* = 131)

### Variants Overrepresented in African American Children with Severe Sepsis

**Fig. 5:**
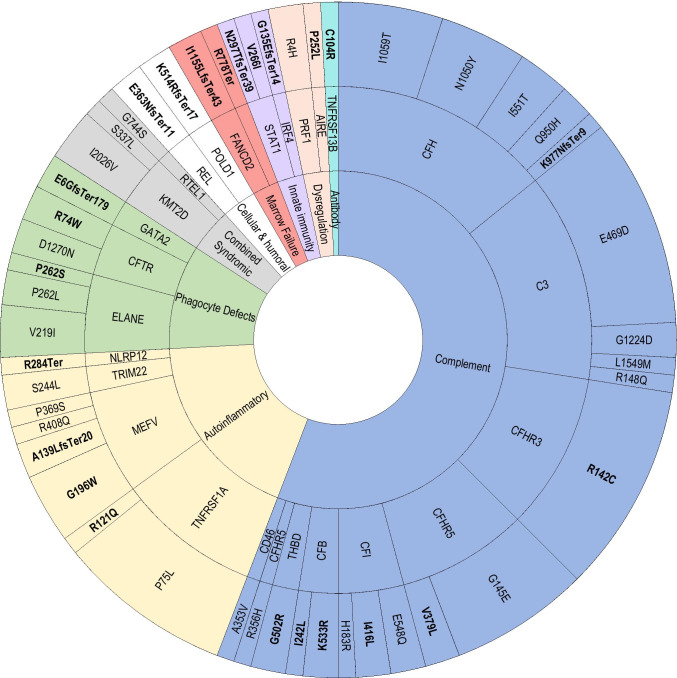
Genomic landscape of inborn errors of immunity in African American children with severe pediatric sepsis. This figure displays every pathogenic or potentially pathogenic variant identified in African American children with severe sepsis. From the inner- to the outermost ring, it indicates the variants’ International Union of Immunologic Societies Classification, the human disease where it has been previously identified, the gene locus affected, and finally, the specific allele change. Representatives of all IUIS disease classes were identified in the cohort including from the most to the least common: complement deficiencies (complement, blue), autoinflammatory disorders (autoinflammatory, yellow), defects of phagocyte number and function (phagocyte defects, green), combined immunodeficiency with associated or syndromic features (syndromic features, gray), immunodeficiencies affecting cellular and humoral immunity (T and B cell, white), bone marrow failure disorders (marrow failure, red), defects of innate and intrinsic immunity (innate immunity, purple), congenital diseases of immune dysregulation (immune dysregulation, peach), and predominantly antibody deficiencies (antibody, teal). Variants assigned DM in the HGMD database are shown in bold, while those with DM? designations are shown in plain font face. The wedge size allotted to an individual variant, gene, disease, and immune disorder classification is proportional to the relative frequency of its observation in the dataset

After demonstrating that African American children had increased odds of variant identification, we sought to identify specific contributing variants. In African American children with severe sepsis, complement variants represented a higher proportion of variants than in the complete cohort (Fig. 5, *N* = 38/70 vs. Figure 3, *N* = 92/330, *p* < 0.0002). When comparing African American to non-African American children in our cohort, the activating convertase variant *C3* (c.1407G > C; p.Glu469Asp), complement regulatory variants *CFHR3* (c.424C > T; p.Arg142Cys) and *CFHR5* (c.434G > A; p.Gly145Glu), and *TNFRSF1A* (c.224C > T; p.Pro75Leu) an autoinflammation variant associated with increased NF-*k*B p65 activity and IL-8 secretion [31] were overrepresented (Table 2). Next, we compared MAF in African American children with sepsis to individuals of African descent in gnomAD, highlighting potential predisposing factors for severe sepsis among groups of similar ancestral backgrounds. While limited by small numbers, we saw that *C3* (c. 443G > A; p.Arg148Gln) and *CFH* (c.2850G > T; p.Gln950His) were both more common than expected in African American children with sepsis (Table 2).

## Discussion

In our severe sepsis cohort, over half of the children had at least one IEI-linked variant. While overestimating immunodeficiency, we suggest this accurately estimates the proportion of children with genetic risk for immune dysfunction during infection as evidenced by increased odds of positive blood or urine culture, hyperferritinemia, thrombocytopenia, lymphopenia, elevated CRP, and increased ECMO use. While septic episodes may rarely identify children with immunodeficiency, future studies of the impact of these variants on host response to infection may provide mechanistic insight into the sepsis-related dysregulated immune response and inform mechanism-based immunomodulatory therapy. This may be especially true in tertiary intensive care units in resource-rich settings, as their patients may represent a significantly enriched population because of immunization practices, early and aggressive antibiotic treatment, and low rates of endemic infection.

In a recent report, Borghesi et al. used WES to identify immunodeficiency variants in 20% of previously healthy children with positive blood cultures, predominantly described as variants of uncertain significance [8]. While these study participants had bacteremia, only 38% were admitted to ICU, and less than half had organ dysfunction, a necessary criterion for diagnosis of severe sepsis. Their variant filtering included use of an in-house database, in silico modeling, and private literature search contributing to differences in prevalence estimates. Still, both studies suggest that variation in immunodeficiency genes is associated with pediatric sepsis. Additionally, in our study of severe pediatric sepsis, we were further able to associate genetic findings with infection site, inflammatory response, and organ support with ECMO. Our pediatric findings are consistent with our previous report that six of six adults with extreme hyperferritinemic sepsis had pathogenic and potentially pathogenic IEI variants [32].

In all children with severe sepsis and in septic African American children specifically, complement variants were frequent. As part of innate immunity, complement functions in early pathogen response. Inactivating variants increase susceptibility to bacteria [4, 33] and can be selected against in settings of endemic infection [34]. However, improved pathogen clearance may increase inflammation and thrombotic microangiopathy [35]. In this regard, *C3* c.1407G > C and *CFHR3* c.424C > T, the most common variants in our cohort, have been reported as causal for aHUS [27, 29] and were statistically overrepresented in comparison to gnomAD. In addition to their reports in aHUS, other complement variants have also been observed in additional thrombotic phenotypes [36] including recurrent pregnancy loss (*C3* c.2203C > T) [37], HELLP syndrome (*CD46* c.1058C > T, *CD46* c.38C > T) [38, 39], and drug-induced thrombotic microangiopathy (C*FH* c.2850G > T, *CFH* c.3148A > T) [28]. Our observation that children with complement variants had increased odds of thrombocytopenia, hyperferritinemia, and having a CRP > 10 mg/dl suggests that these variants convey risk for microangiopathy during episodes of severe sepsis.

When considering variant frequency, it is important to emphasize ancestry-specific differences. For *C3* c.1407G > C, *CFHR3* c.424C > T, *CFHR5* c.434G > A, and *TNFRSF1A* c.224C > T, a TNF*α* receptor that directly activates complement signaling [40], overrepresentation may be partially explained by their frequency in African Americans. Still, complement overrepresentation is not completely explained by population stratification, as when African American children with severe sepsis are compared to African gnomAD participants, *C3* c.443G > A and *CFH* c.2850G > T remained overrepresented. Acknowledging that African gnomAD participants are an imperfect control, previous reports of variants’ disease impact emphasize complement’s biologic relevance in sepsis pathobiology (Table S2). These findings lead us to hypothesize that observed differences in pediatric sepsis outcomes that associate with ancestry may be impacted by complement genetics and emphasize the need for enrollment of diverse cohorts in future genetic studies of severe pediatric sepsis.

The second most common functional group was autoinflammation variants. *NLRP3* variants cause the cryopyrin-associated periodic syndromes including familial cold inflammatory syndrome, Muckle–Wells syndrome, and neonatal-onset multisystem inflammatory disorder (NOMID). These disorders are monogenic inflammasomopathies inherited in an autosomal dominant pattern with incomplete penetrance. The specific *NLRP3* p.Gln705Lys variant leads to constitutive hyperactivation with increased IL-1*β* and IL-18 synthesis [41] that associates with the severity of acute phase response [42]. We also commonly observed *MEFV* variants, previously reported in the familial Mediterranean fever that has been associated with an exaggerated inflammatory response to infection with larger increases in WBC count, ESR and LDH levels, more pronounced tachycardia, and hypotension [43].

*IRF3* (c.829G > A; p.Ala277Thr) variants were also encountered more commonly in the pediatric sepsis cohort than expected (adjusted *p* = 0.013). Heterozygous *IRF3* variants (c.829G > A; p.Ala277Thr) have been described in herpes simplex encephalitis and peripheral blood mononuclear cells isolated from individuals with *IRF3* c. 829G > A have significantly lower CXCL10 and IFN-*β* levels following poly(I:C), HSV-1 (a DNA virus) and RNA virus exposure, suggesting impaired antiviral response [30]. Additionally, other *IRF3* variants are overrepresented among individuals with life-threatening SARS-CoV-2 infection, where they are hypothesized to impair viral clearance [44]. As viral infections are a common cause of sepsis in children, this leads us to hypothesize that genetic interferon pathway variation may associate with risk for viral sepsis in general; however, as the absolute number of children with *IRF3* variants in the cohort was small (*N* = 9), we did not detect an increased rate of viral sepsis.

Other key findings include, that if offered in the PICU, genetic testing for immunologic disease is agreed to by 95% of parents of children with sepsis. The acceptability of genetic testing is important in light of questions surrounding patient and family preferences. We also found an insufficient sampling of DNA was common in children with lymphopenia, suggesting a role for buccal mucosa, saliva, or uroepithelial cell sampling techniques. This is of considerable importance, as sepsis patients with lymphopenia are at greater risk of morbidity and mortality [18]. Children with malignancy are also commonly lymphopenic, limiting the generalizability of our findings to this common sepsis subpopulation.

Our study’s main limitation is that while the presence of a pathogenic or potentially pathogenic variant in a disease-consistent inheritance pattern is remarkable, it cannot be equated with immunodeficiency. While candidate variants were restricted to those with prior associations with human disease, literature-based classification is likely to misclassify a portion of variants and cannot be equated to clinical sequencing. We used the HGMD professional database for variant assignment, a resource curated to minimize false negatives that consequently increases false positives in the assignment of pathogenicity. Additionally, it is known that even well-established pathogenic variants do not always cause disease due to variable penetrance, expressivity, epistasis, gene–gene interactions, and environmental factors [10]. Therefore, even pathogenic (DM) variants for autosomal dominant conditions represent potential rather than confirmed immunodeficiencies. Additionally, the lack of long-term follow-up, functional immunologic testing, and characterization of dysmorphic features limits our ability to correlate our genetic findings to phenotype. However, the ability of our study to identify associations between IEI and types of infection, inflammatory response, and need for extracorporeal therapy despite the noise introduced by incorrect assignment, emphasizes the functional relevance of IEI in pediatric sepsis, while acknowledging that this analysis cannot be interpreted as evidence for individual variant pathogenicity. The enrollment of children with only severe sepsis and organ failure also limits the generalizability of our findings to septic children without organ failure. However, those with organ failure disproportionately represent sepsis-related morbidity and mortality, emphasizing a need for further study of the impact of IEI variants on host–pathogen interactions, the dysregulated host response, and the need for organ support therapy once the infection is established. Other notable limitations include the 10 cases of the potential autosomal recessive biallelic disease, where WES is unable to differentiate *cis* from *trans* variants in the absence of parental sampling. WES also fails to identify regulatory, structural, and copy number variants. A future study is needed to determine the impact of these variants on predisposition to infection, dysregulated host response, illness severity, and recurrence risk between ancestry groups and among individuals with shared genetic risk.

## Conclusions

In conclusion, genetic variation previously linked to inborn errors of immunity is common in our pediatric severe sepsis cohort. These variants were associated with bacteremia, urinary tract infection, laboratory markers of inflammation, and requirement for extracorporeal membrane oxygenation. This suggests that the evaluation of children with severe sepsis is warranted by clinical geneticists to screen for primary immunodeficiencies. A future study of heritable immunologic differences in children with severe sepsis may enable a genome-driven approach to the dysregulated host response.

## Supplementary Information

Below is the link to the electronic supplementary material.

Supplementary file1 (XLSX 35 KB)

Supplementary file2 (DOCX 944 KB)

Supplementary file3 (XLSX 65 KB)

Supplementary file4 (XLS 45 KB)

Supplementary file5 (PDF 49 KB)

Supplementary file6 (PDF 46 KB)

Supplementary file7 (XLSX 40 KB)

## Data Availability

All data used in this analysis are available in the main and extended tables.
