# Supplementary material for: Prevalence of Pathogenic and Potentially Pathogenic Inborn Error of Immunity Associated Variants in Children with Severe Sepsis
Source: J Clin Immunol. 2022 Jan 1;42(2):350–64. doi: 10.1007/s10875-021-01183-4 (PMC8720168; doi:10.1007/s10875-021-01183-4)
Supplement: Supplementary file 2 — Supplementary file2 (DOCX 944 KB) [file 10875_2021_1183_MOESM2_ESM.docx]

| **Gene** | **HGVS c** | **HGVS p** | **PMID** |
| --- | --- | --- | --- |
| *ACD* | c.1285_1286insC | p.Ser429ThrfsTer10 | NA |
| *AIRE* | c.1411C>T | p.Arg471Cys | [1–6] |
| *AIRE* | c.755C>T | p.Pro252Leu | [7] |
| *AIRE* | c.901G>A | p.Val301Met | [8,9] |
| *C3* | c.1407G>C | p.Glu469Asp | [10–13] |
| *C3* | c.2203C>T | p.Arg735Trp | [14–16] |
| *C3* | c.26T>C | p.Leu9Pro | [11] |
| *C3* | c.3671G>A | p.Gly1224Asp | [11] |
| *C3* | c.443G>A | p.Arg148Gln | [17] |
| *C3* | c.4645C>A | p.Leu1549Met | [10] |
| *C3* | c.4855A>C | p.Ser1619Arg | [11,14,18,19] |
| *CARD14* | c.1772C>T | p.Thr591Met | [20,21] |
| *CARD14* | c.205C>T | p.Arg69Trp | [22] |
| *CASP10* | c.1216A>T | p.Ile406Leu | [23,24] |
| *CD46* | c.1058C>T | p.Ala353Val | [13,16,25–28] |
| *CD46* | c.287-2A>G |  | [16,29–34] |
| *CD46* | c.38C>T | p.Ser13Phe | [35,36] |
| *CFB* | c.1598A>G | p.Lys533Arg | [30,37–41] |
| *CFB* | c.1697A>C | p.Glu566Ala | [15,40,42–44] |
| *CFB* | c.724A>C | p.Ile242Leu | [40,45–47] |
| *CFH* | c.1652T>C | p.Ile551Thr | [11] |
| *CFH* | c.2850G>T | p.Gln950His | [11,30,48–54] |
| *CFH* | c.2931delA | p.Lys977AsnfsTer9 | Novel |
| *CFH* | c.3148A>T | p.Asn1050Tyr | [11,15,50,51,55] |
| *CFH* | c.3176T>C | p.Ile1059Thr | [11,56] |
| *CFH* | c.7C>G | p.Leu3Val | [11] |
| *CFH* | c.920dup | p.Cys309MetfsTer18 | Novel |
| *CFHR3* | c.424C>T | p.Arg142Cys | [36] |
| *CFHR3* | c.424C>T | p.Arg142Cys | [36] |
| *CFHR4* | c.1177+2T>C |  | Novel |
| *CFHR5* | c.1067G>A | p.Arg356His | [57] |
| *CFHR5* | c.1135G>C | p.Val379Leu | [45] |
| *CFHR5* | c.434G>A | p.Gly145Glu | [36] |
| *CFHR5* | c.485_486dup | p.Glu163LysfsTer10 | Novel |
| *CFHR5* | c.61del | p.Thr21HisfsTer50 | Novel |
| *CFI* | c.1246A>C | p.Ile416Leu | [26,47,58–62] |
| *CFI* | c.1322A>G | p.Lys441Arg | [11,63,64] |
| *CFI* | c.1642G>C | p.Glu548Gln | [11,30,65,66] |
| *CFI* | c.548A>G | p.His183Arg | [16,47,62,67] |
| *CFI* | c.782G>A | p.Gly261Asp | [11,16,68–70] |
| *CFTR* | c.220C>T | p.Arg74Trp | [71–75] |
| *CFTR* | c.221G>A | p.Arg74Gln | [76–78] |
| *CFTR* | c.3808G>A | p.Asp1270Asn | [74,79,80] |
| *CFTR* | c.890G>A | p.Arg297Gln | [81–84] |
| *CHD7* | c.127A>G | p.Ile43Val | [85] |
| *CHD7* | c.2230G>A | p.Gly744Ser | [86,87] |
| *CHD7* | c.2413delA | p.Ile805LeufsTer2 | Novel |
| *CTLA4* | c.326G>A | p.Gly109Glu | [88] |
| *ELANE* | c.428G>A | p.Arg143His | [89] |
| *ELANE* | c.655G>A | p.Val219Ile | [90] |
| *ELANE* | c.770C>T | p.Pro257Leu | [91,92] |
| *ELANE* | c.784C>T | p.Pro262Ser | [93] |
| *ERBB2IP* | c.1303delG | p.Asp435MetfsTer46 | Novel |
| *ERBB2IP* | c.3053delA | p.Asn1018IlefsTer11 | Novel |
| *FANCD2* | c.3463delA | p.Ile1155LeufsTer43 | Novel |
| *FANCG* | c.890C>T | p.Thr297Ile | [94,95] |
| *FAS* | c.580G>A | p.Glu194Lys | [96,97] |
| *GATA2* | c.16dup | p.Glu6GlyfsTer179 | Novel |
| *IL17RA* | c.1188delG | p.Lys397AsnfsTer30 | Novel |
| *IL2RG* | c.115+5G>A |  | [98] |
| *IRF3* | c.829G>A | p.Ala277Thr | [99] |
| *IRF4* | c.402_403delAGinsGT | p.Gly135Ter | Novel |
| *ITGB2* | c.1323del | p.Leu442PhefsTer87 | Novel |
| *ITGB2* | c.821dupA | p.Leu275AlafsTer39 | Novel |
| *KMT2A* | c.10850T>C | p.Leu3617Pro | [100] |
| *KMT2D* | c.1010C>T | p.Ser337Leu | [101,102] |
| *KMT2D* | c.10192A>G | p.Met3398Val | [102,103] |
| *KMT2D* | c.3392C>T | p.Pro1131Leu | [102,103] |
| *KMT2D* | c.3572C>T | p.Pro1191Leu | [102,103] |
| *KMT2D* | c.5477G>T | p.Gly1826Val | [102] |
| *KMT2D* | c.6076A>G | p.Ile2026Val | [102] |
| *KMT2D* | c.6437C>T | p.Pro2146Leu | [102] |
| *KMT2D* | c.6955dup | p.Leu2319ProfsTer7 | Novel |
| *MEFV* | c.1105C>T | p.Pro369Ser | [104–108] |
| *MEFV* | c.1223G>A | p.Arg408Gln | [109–112] |
| *MEFV* | c.1772T>C | p.Ile591Thr | [113–115] |
| *MEFV* | c.2084A>G | p.Lys695Arg | [116–119] |
| *MEFV* | c.2230G>T | p.Ala744Ser | [117,120,121] |
| *MEFV* | c.289C>A | p.Gln97Lys | [122] |
| *MEFV* | c.329T>C | p.Leu110Pro | [123–128] |
| *MEFV* | c.414del | p.Ala139LeufsTer20 | Novel |
| *MEFV* | c.586G>T | p.Gly196Trp | [129] |
| *NLRP12* | c.850C>T | p.Arg284Ter | [130,131] |
| *NLRP12* | c.910C>T | p.His304Tyr | [132–135] |
| *NLRP3* | c.2113C>A | p.Gln705Lys | [136–140] |
| *NLRP3* | c.2182A>G | p.Ser728Gly | [141] |
| *NLRP3* | c.598G>A | p.Val200Met | [138,142–146] |
| *NOD2* | c.2264C>T | p.Ala755Val | [147] |
| *POLD1* | c.1539delG | p.Lys514ArgfsTer17 | Novel |
| *POLR3C* | c.251G>A | p.Arg84Gln | [148] |
| *PRF1* | c.11G>A | p.Arg4His | [149] |
| *PSTPIP1* | c.1213C>T | p.Arg405Cys | [150,151] |
| *REL* | c.1086delA | p.Glu363AsnfsTer11 | Novel |
| *RTEL1* | c.2332C>T | p.Arg778Ter | Novel |
| *RTEL1* | c.2879dupG | p.Leu962ProfsTer4 | Novel |
| *SKIV2L* | c.3055dupC | p.Arg1019ProfsTer36 | Novel |
| *SLC39A7* | c.170del | p.Gly57AlafsTer88 | Novel |
| *SPINK5* | c.1658dupT | p.Glu554ArgfsTer3 | Novel |
| *SRP72* | c.20_21insT | p.Val9GlyfsTer9 | Novel |
| *STAT1* | c.796G>A | p.Val266Ile | [99,152–154] |
| *STAT1* | c.890delA | p.Asn297ThrfsTer39 | Novel |
| *TAP1* | c.147_148insA | p.Pro50ThrfsTer14 | Novel |
| *TBX1* | c.1452del | p.Ala485ArgfsTer? | Novel |
| *TCF3* | c.1921dupC | p.His641ProfsTer61 | Novel |
| *TCF3* | c.302A>G | p.Lys101Arg | [155] |
| *TERT* | c.1234C>T | p.His412Tyr | [68,156,157] |
| *TERT* | c.1323_1325del | p.Glu441del | [158,159] |
| *TERT* | c.336del | p.Glu113ArgfsTer15 | Novel |
| *TERT* | c.560del | p.Pro187ArgfsTer164 | Novel |
| *TERT* | c.604G>A | p.Ala202Thr | [156,159–161] |
| *THBD* | c.1208G>A | p.Arg403Lys | [162,163] |
| *THBD* | c.1456G>T | p.Asp486Tyr | [162,164–166] |
| *THBD* | c.1504G>C | p.Gly502Arg | [56] |
| *TICAM1* | c.1702G>A | p.Ala568Thr | [99] |
| *TINF2* | c.734C>A | p.Ser245Tyr | [68,167,168] |
| *TNFRSF13B* | c.260T>A | p.Ile87Asn | [169–172] |
| *TNFRSF13B* | c.310T>C | p.Cys104Arg | [173–178] |
| *TNFRSF13B* | c.542C>A | p.Ala181Glu | [173,179–181] |
| *TNFRSF1A* | c.224C>T | p.Pro75Leu | [182–185] |
| *TNFRSF1A* | c.362G>A | p.Arg121Gln | [182,183,185–190] |
| *TRIM22* | c.731C>T | p.Ser244Leu | [191] |
| *TTC37* | c.2448delA | p.Ala817GlnfsTer2 | Novel |
| *UNC13D* | c.175G>A | p.Ala59Thr | [192,193] |
| *UNC13D* | c.2341G>A | p.Val781Ile | [193] |
| *UNC13D* | c.2542A>C | p.Ile848Leu | [13,193] |
| *UNC13D* | c.2553+5C>G |  | [194–197] |
| *UNC13D* | c.2782C>T | p.Arg928Cys | [13,192,193] |
| *UNC13D* | c.2983G>C | p.Ala995Pro | [13,192] |
| *UNC13D* | c.460C>T | p.Arg154Trp | [198] |
| *XIAP* | c.738delT | p.Asp247IlefsTer46 | Novel |

1. Cervato S, Morlin L, Albergoni MP, Masiero S, Greggio N, Meossi C, et al. AIRE gene mutations and autoantibodies to interferon omega in patients with chronic hypoparathyroidism without APECED. Clinical Endocrinology [Internet]. Clin Endocrinol (Oxf); 2010 [cited 2021 Jul 1];73:630–6. Available from: https://pubmed.ncbi.nlm.nih.gov/20718774/

2. Tóth B, Wolff ASB, Halász Z, Tar A, Szüts P, Ilyés I, et al. Novel sequence variation of AIRE and detection of interferon-ω antibodies in early infancy. Clinical Endocrinology [Internet]. Clin Endocrinol (Oxf); 2010 [cited 2021 Jul 1];72:641–7. Available from: https://pubmed.ncbi.nlm.nih.gov/19863576/

3. Fierabracci A. The role of heterozygous mutations of the autoimmune regulator gene (AIRE) in non-APECED autoimmunity: A comment on recent findings [Internet]. Clinical Endocrinology. Clin Endocrinol (Oxf); 2011 [cited 2021 Jul 1]. p. 532–3. Available from: https://pubmed.ncbi.nlm.nih.gov/21070315/

4. Betterle C, Scarpa R, Garelli S, Morlin L, Lazzarotto F, Presotto F, et al. Addison’s disease: A survey on 633 patients in Padova. European Journal of Endocrinology [Internet]. BioScientifica Ltd.; 2013 [cited 2021 Jul 1];169:773–84. Available from: https://pubmed.ncbi.nlm.nih.gov/24014553/

5. Resende E, Gόmez GN, Nascimento M, Loidi L, Fiaño RS, Rodrίguez PC, et al. Precocious presentation of autoimmune polyglandular syndrome type 2 associated with an AIRE mutation. Hormones [Internet]. Hellenic Endocrine Society; 2015 [cited 2021 Jul 1];14:312–6. Available from: https://pubmed.ncbi.nlm.nih.gov/25402387/

6. Tsai SL, Green J, Metherell LA, Curtis F, Fernandez B, Healey A, et al. Primary adrenocortical insufficiency case series: Genetic etiologies more common than expected. Hormone Research in Paediatrics [Internet]. S. Karger AG; 2016 [cited 2021 Jul 1];85:35–42. Available from: https://pubmed.ncbi.nlm.nih.gov/26650942/

7. Meloni A, Perniola R, Faà V, Corvaglia E, Cao A, Rosatelli MC. Delineation of the molecular defects in the AIRE gene in autoimmune polyendocrinopathy-candidiasis-ectodermal dystrophy patients from Southern Italy. Journal of Clinical Endocrinology and Metabolism [Internet]. Endocrine Society; 2002 [cited 2021 Jul 1];87:841–6. Available from: https://pubmed.ncbi.nlm.nih.gov/11836330/

8. Oftedal BE, Hellesen A, Erichsen MM, Bratland E, Vardi A, Perheentupa J, et al. Dominant Mutations in the Autoimmune Regulator AIRE Are Associated with Common Organ-Specific Autoimmune Diseases. Immunity [Internet]. Cell Press; 2015 [cited 2021 Jul 1];42:1185–96. Available from: https://pubmed.ncbi.nlm.nih.gov/26084028/

9. Söderbergh A, Rorsman F, Halonen M, Ekwall O, Björses P, Kämpe O, et al. Autoantibodies against Aromatic l-Amino Acid Decarboxylase Identifies a Subgroup of Patients with Addison’s Disease1. The Journal of Clinical Endocrinology & Metabolism [Internet]. The Endocrine Society; 2000 [cited 2021 Jul 1];85:460–3. Available from: https://pubmed.ncbi.nlm.nih.gov/10634424/

10. Schramm EC, Roumenina LT, Rybkine T, Chauvet S, Vieira-Martins P, Hue C, et al. Mapping interactions between complement C3 and regulators using mutations in atypical hemolytic uremic syndrome. Blood. American Society of Hematology; 2015;125:2359–69.

11. Geerlings MJ, Volokhina EB, de Jong EK, van de Kar N, Pauper M, Hoyng CB, et al. Genotype-phenotype correlations of low-frequency variants in the complement system in renal disease and age-related macular degeneration. Clinical Genetics [Internet]. Blackwell Publishing Ltd; 2018 [cited 2020 Apr 25];94:330–8. Available from: http://doi.wiley.com/10.1111/cge.13392

12. Fidalgo T, Martinho P, Pinto CS, Oliveira AC, Salvado R, Borràs N, et al. Combined study of ADAMTS13 and complement genes in the diagnosis of thrombotic microangiopathies using next-generation sequencing. Research and Practice in Thrombosis and Haemostasis [Internet]. Blackwell Publishing Ltd; 2017 [cited 2021 Jul 1];1:69–80. Available from: https://pubmed.ncbi.nlm.nih.gov/30046676/

13. Kernan KF, Ghaloul-Gonzalez L, Shakoory B, Kellum JA, Angus DC, Carcillo JA. Adults with septic shock and extreme hyperferritinemia exhibit pathogenic immune variation. Genes and immunity [Internet]. 2018 [cited 2018 Sep 6]; Available from: http://www.ncbi.nlm.nih.gov/pubmed/29977033

14. Mohlin FC, Gros P, Mercier E, Gris JCR, Blom AM. Analysis of C3 gene variants in patients with idiopathic recurrent spontaneous pregnancy loss. Frontiers in Immunology. Frontiers Media S.A.; 2018;9.

15. Bu F, Zhang Y, Wang K, Borsa NG, Jones MB, Taylor AO, et al. Genetic analysis of 400 patients refines understanding and implicates a new gene in atypical hemolytic uremic syndrome. Journal of the American Society of Nephrology [Internet]. American Society of Nephrology; 2018 [cited 2021 Jul 1];29:2809–19. Available from: https://pubmed.ncbi.nlm.nih.gov/30377230/

16. Bresin E, Rurali E, Caprioli J, Sanchez-Corral P, Fremeaux-Bacchi V, de Cordoba SR, et al. Combined complement gene mutations in atypical hemolytic uremic syndrome influence clinical phenotype. Journal of the American Society of Nephrology [Internet]. J Am Soc Nephrol; 2013 [cited 2021 Jul 1];24:475–86. Available from: https://pubmed.ncbi.nlm.nih.gov/23431077/

17. High-Throughput Genetic Testing for Thrombotic Microangiopathies and C3 Glomerulopathies.

18. Feng S, Eyler SJ, Zhang Y, Maga T, Nester CM, Kroll MH, et al. Partial ADAMTS13 deficiency in atypical hemolytic uremic syndrome. Blood [Internet]. 2013 [cited 2018 May 29];122:1487–93. Available from: http://www.bloodjournal.org/cgi/doi/10.1182/blood-2013-03-492421

19. Bu F, Maga T, Meyer NC, Wang K, Thomas CP, Nester CM, et al. Comprehensive genetic analysis of complement and coagulation genes in atypical hemolytic uremic syndrome. Journal of the American Society of Nephrology [Internet]. J Am Soc Nephrol; 2014 [cited 2021 Jul 1];25:55–64. Available from: https://pubmed.ncbi.nlm.nih.gov/24029428/

20. Qin P, Zhang Q, Chen M, Fu X, Wang C, Wang Z, et al. Variant Analysis of CARD14 in a Chinese Han Population with Psoriasis Vulgaris and Generalized Pustular Psoriasis [Internet]. Journal of Investigative Dermatology. Nature Publishing Group; 2014 [cited 2021 Jul 1]. p. 2994–6. Available from: https://pubmed.ncbi.nlm.nih.gov/24999592/

21. Li L, You J, Fu X, Wang Z, Sun Y, Liu H, et al. Variants of CARD14 are predisposing factors for generalized pustular psoriasis (GPP) with psoriasis vulgaris but not for GPP alone in a Chinese population [Internet]. British Journal of Dermatology. Blackwell Publishing Ltd; 2019 [cited 2021 Jul 1]. p. 425–6. Available from: https://pubmed.ncbi.nlm.nih.gov/30387497/

22. Ammar M, Jordan CT, Cao L, Lim E, Bouchlaka Souissi C, Jrad A, et al. CARD14 alterations in Tunisian patients with psoriasis and further characterization in European cohorts. British Journal of Dermatology [Internet]. Blackwell Publishing Ltd; 2016 [cited 2021 Jul 1];174:330–7. Available from: https://pubmed.ncbi.nlm.nih.gov/26358359/

23. Zhu S, Hsu AP, Vacek MM, Zheng L, Schäffer AA, Dale JK, et al. Genetic alterations in caspase-10 may be causative or protective in autoimmune lymphoproliferative syndrome. Human Genetics [Internet]. Hum Genet; 2006 [cited 2021 Jul 1];119:284–94. Available from: https://pubmed.ncbi.nlm.nih.gov/16446975/

24. Tripodi SI, Mazza C, Moratto D, Ramenghi U, Caorsi R, Gattorno M, et al. Atypical presentation of autoimmune lymphoproliferative syndrome due to CASP10 mutation. Immunology Letters [Internet]. Elsevier B.V.; 2016 [cited 2021 Jul 1];177:22–4. Available from: https://pubmed.ncbi.nlm.nih.gov/27378136/

25. Caprioli J, Noris M, Brioschi S, Pianetti G, Castelletti F, Bettinaglio P, et al. Genetics of HUS: The impact of MCP, CFH, and IF mutations on clinical presentation, response to treatment, and outcome. Blood [Internet]. Blood; 2006 [cited 2021 Jul 1];108:1267–79. Available from: https://pubmed.ncbi.nlm.nih.gov/16621965/

26. Salmon JE, Heuser C, Triebwasser M, Liszewski MK, Kavanagh D, Roumenina L, et al. Mutations in complement regulatory proteins predispose to preeclampsia: A genetic analysis of the PROMISSE cohort. PLoS Medicine [Internet]. PLoS Med; 2011 [cited 2021 Jul 1];8. Available from: https://pubmed.ncbi.nlm.nih.gov/21445332/

27. Sartz L, Olin AI, Kristoffersson A-C, Ståhl A, Johansson ME, Westman K, et al. A Novel C3 Mutation Causing Increased Formation of the C3 Convertase in Familial Atypical Hemolytic Uremic Syndrome. The Journal of Immunology [Internet]. The American Association of Immunologists; 2012 [cited 2021 Jul 1];188:2030–7. Available from: https://pubmed.ncbi.nlm.nih.gov/22250080/

28. Shoemaker LD, Clark MJ, Patwardhan A, Chandratillake G, Garcia S, Chen R, et al. Disease variant landscape of a large multiethnic population of moyamoya patients by exome sequencing. G3: Genes, Genomes, Genetics [Internet]. Genetics Society of America; 2016 [cited 2021 Jul 1];6:41–9. Available from: https://pubmed.ncbi.nlm.nih.gov/26530418/

29. Caprioli J, Noris M, Brioschi S, Pianetti G, Castelletti F, Bettinaglio P, et al. Genetics of HUS: the impact of MCP, CFH, and IF mutations on clinical presentation, response to treatment, and outcome. Blood. The American Society of Hematology; 2006;108:1267–79.

30. Fidalgo T, Martinho P, Pinto CS, Oliveira AC, Salvado R, Borràs N, et al. Combined study of ADAMTS13 and complement genes in the diagnosis of thrombotic microangiopathies using next-generation sequencing. Research and Practice in Thrombosis and Haemostasis [Internet]. Blackwell Publishing Ltd; 2017 [cited 2021 Jul 1];1:69–80. Available from: https://pubmed.ncbi.nlm.nih.gov/30046676/

31. Phillips EH, Westwood JP, Brocklebank V, Wong EKS, Tellez JO, Marchbank KJ, et al. The role of ADAMTS-13 activity and complement mutational analysis in differentiating acute thrombotic microangiopathies. Journal of Thrombosis and Haemostasis [Internet]. Blackwell Publishing Ltd; 2016 [cited 2021 Jul 1];14:175–85. Available from: https://pubmed.ncbi.nlm.nih.gov/26559391/

32. Xiong HY, Alipanahi B, Lee LJ, Bretschneider H, Merico D, Yuen RKC, et al. The human splicing code reveals new insights into the genetic determinants of disease. Science [Internet]. American Association for the Advancement of Science; 2015 [cited 2021 Jul 1];347. Available from: https://pubmed.ncbi.nlm.nih.gov/25525159/

33. Pabst WL, Neuhaus TJ, Nef S, Bresin E, Zingg-Schenk A, Spartà G. Successful long-term outcome after renal transplantation in a patient with atypical haemolytic uremic syndrome with combined membrane cofactor protein CD46 and complement factor i mutations. Pediatric Nephrology [Internet]. Pediatr Nephrol; 2013 [cited 2021 Jul 1];28:1141–4. Available from: https://pubmed.ncbi.nlm.nih.gov/23519521/

34. Marini SC, Gomes M, Guilherme R, Carda JP, Pinto CS, Fidalgo T, et al. Atypical hemolytic-uremic syndrome: Recurrent phenotypic expression of a patient with MCP gene mutation combined with risk haplotypes. Blood Coagulation and Fibrinolysis [Internet]. Lippincott Williams and Wilkins; 2019 [cited 2021 Jul 1];30:68–70. Available from: https://pubmed.ncbi.nlm.nih.gov/30676336/

35. Crovetto F, Borsa N, Acaia B, Nishimura C, Frees K, Smith RJH, et al. The genetics of the alternative pathway of complement in the pathogenesis of HELLP syndrome. Journal of Maternal-Fetal and Neonatal Medicine [Internet]. J Matern Fetal Neonatal Med; 2012 [cited 2021 Feb 3];25:2322–5. Available from: https://pubmed.ncbi.nlm.nih.gov/22594569/

36. Zhang T, Lu J, Liang S, Chen D, Zhang H, Zeng C, et al. Comprehensive analysis of complement genes in patients with atypical hemolytic uremic syndrome. American Journal of Nephrology [Internet]. S. Karger AG; 2016 [cited 2020 Dec 9];43:160–9. Available from: https://pubmed.ncbi.nlm.nih.gov/27064621/

37. Thergaonkar RW, Narang A, Gurjar BS, Tiwari P, Puraswani M, Saini H, et al. Targeted exome sequencing in anti-factor H antibody negative HUS reveals multiple variations. Clinical and Experimental Nephrology [Internet]. Springer Tokyo; 2018 [cited 2021 Jul 1];22:653–60. Available from: https://pubmed.ncbi.nlm.nih.gov/28939980/

38. Noronha N, Costa FD, Dias A, Dinis A. Complement factor B mutation-associated aHUS and myocardial infarction. BMJ Case Reports [Internet]. BMJ Publishing Group; 2017 [cited 2021 Jul 1];2017. Available from: https://pubmed.ncbi.nlm.nih.gov/28710236/

39. Zhu Z, Chen H, Gill R, Wang J, Spitalewitz S, Gotlieb V. Diabetic ketoacidosis presenting with atypical hemolytic uremic syndrome associated with a variant of complement factor B in an adult: A case report. Journal of Medical Case Reports [Internet]. BioMed Central; 2016 [cited 2021 Jul 1];10. Available from: https://pubmed.ncbi.nlm.nih.gov/26911616/

40. Marinozzi MC, Vergoz L, Rybkine T, Ngo S, Bettoni S, Pashov A, et al. Complement factor b mutations in atypical hemolytic uremic syndrome-disease-relevant or benign? Journal of the American Society of Nephrology [Internet]. American Society of Nephrology; 2014 [cited 2021 Jul 1];25:2053–65. Available from: https://pubmed.ncbi.nlm.nih.gov/24652797/

41. Tawadrous H, Maga T, Sharma J, Kupferman J, Smith RJH, Schoeneman M. A novel mutation in the Complement Factor B gene (CFB) and atypical hemolytic uremic syndrome. Pediatric Nephrology [Internet]. Pediatr Nephrol; 2010 [cited 2021 Jul 1];25:947–51. Available from: https://pubmed.ncbi.nlm.nih.gov/20108004/

42. Sethi S, Smith RJH, Dillon JJ, Fervenza FC. C3 glomerulonephritis associated with complement factor B mutation [Internet]. American Journal of Kidney Diseases. W.B. Saunders; 2015 [cited 2021 Jul 1]. p. 520–1. Available from: https://pubmed.ncbi.nlm.nih.gov/25532781/

43. Besbas N, Gulhan B, Soylemezoglu O, Ozcakar ZB, Korkmaz E, Hayran M, et al. Turkish pediatric atypical hemolytic uremic syndrome registry: Initial analysis of 146 patients. BMC Nephrology [Internet]. BioMed Central Ltd.; 2017 [cited 2020 Apr 25];18:6. Available from: http://bmcnephrol.biomedcentral.com/articles/10.1186/s12882-016-0420-6

44. Alfakeeh K, Azar M, Alfadhel M, Abdullah AM, Aloudah N, Alsaad KO. Rare genetic variant in the CFB gene presenting as atypical hemolytic uremic syndrome and immune complex diffuse membranoproliferative glomerulonephritis, with crescents, successfully treated with eculizumab. Pediatric Nephrology [Internet]. Springer Verlag; 2017 [cited 2021 Jul 1];32:885–91. Available from: https://pubmed.ncbi.nlm.nih.gov/28210841/

45. Maga TK, Nishimura CJ, Weaver AE, Frees KL, Smith RJH. Mutations in alternative pathway complement proteins in American patients with atypical hemolytic uremic syndrome. Human Mutation. 2010;31.

46. Chonat S, Chandrakasan S, Kalinyak KA, Ingala D, Gruppo R, Kalfa TA. Atypical haemolytic uraemic syndrome in a patient with sickle cell disease, successfully treated with eculizumab [Internet]. British Journal of Haematology. Blackwell Publishing Ltd; 2016 [cited 2021 Jul 1]. p. 744–7. Available from: https://pubmed.ncbi.nlm.nih.gov/27870017/

47. Larsen CP, Wilson JD, Best-Rocha A, Beggs ML, Hennigar RA. Genetic testing of complement and coagulation pathways in patients with severe hypertension and renal microangiopathy. Modern Pathology [Internet]. Nature Publishing Group; 2018 [cited 2021 Jul 1];31:488–94. Available from: https://pubmed.ncbi.nlm.nih.gov/29148534/

48. Caprioli J, Castelletti F, Bucchioni S, Bettinaglio P, Bresin E, Pianetti G, et al. Complement factor H mutations and gene polymorphisms in haemolytic uraemic syndrome: the C-257T, the A2089G and the G2881T polymorphisms are strongly associated with the disease. Human molecular genetics. 2003;12:3385–95.

49. le Quintrec M, Lionet A, Kamar N, Karras A, Barbier S, Buchler M, et al. Complement mutation-associated de novo thrombotic microangiopathy following kidney transplantation. American journal of transplantation : official journal of the American Society of Transplantation and the American Society of Transplant Surgeons [Internet]. 2008 [cited 2018 Aug 16];8:1694–701. Available from: http://doi.wiley.com/10.1111/j.1600-6143.2008.02297.x

50. Chapin J, Eyler S, Smith R, Tsai HM, Laurence J. Complement factor H mutations are present in ADAMTS13-deficient, ticlopidine-associated thrombotic microangiopathies. [Internet]. Blood. Blood; 2013 [cited 2021 Feb 3]. p. 4012–3. Available from: https://pubmed.ncbi.nlm.nih.gov/23660864/

51. Besbas N, Gulhan B, Soylemezoglu O, Ozcakar ZB, Korkmaz E, Hayran M, et al. Turkish pediatric atypical hemolytic uremic syndrome registry: Initial analysis of 146 patients. BMC Nephrology [Internet]. BioMed Central Ltd.; 2017 [cited 2021 Jul 1];18. Available from: https://pubmed.ncbi.nlm.nih.gov/28056875/

52. Mohlin FC, Nilsson SC, Levart TK, Golubovic E, Rusai K, Müller-Sacherer T, et al. Functional characterization of two novel non-synonymous alterations in CD46 and a Q950H change in factor H found in atypical hemolytic uremic syndrome patients. Molecular Immunology [Internet]. Elsevier Ltd; 2015 [cited 2021 Jul 1];65:367–76. Available from: https://pubmed.ncbi.nlm.nih.gov/25733390/

53. Neumann HPH, Salzmann M, Bohnert-Iwan B, Mannuelian T, Skerka C, Lenk D, et al. Haemolytic uraemic syndrome and mutations of the factor H gene: A registry-based study of German speaking countries. Journal of Medical Genetics [Internet]. BMJ Publishing Group; 2003 [cited 2021 Jul 1];40:676–81. Available from: https://pubmed.ncbi.nlm.nih.gov/12960213/

54. Seaby EG, Gilbert RD, Andreoletti G, Pengelly RJ, Mercer C, Hunt D, et al. Unexpected findings in a child with atypical hemolytic uremic syndrome: An example of how genomics is changing the clinical diagnostic paradigm. Frontiers in Pediatrics [Internet]. Frontiers Media S.A.; 2017 [cited 2021 Jul 1];5. Available from: https://pubmed.ncbi.nlm.nih.gov/28589114/

55. Esparza-Gordillo J, Goicoechea de Jorge E, Buil A, Carreras Berges L, López-Trascasa M, Sánchez-Corral P, et al. Predisposition to atypical hemolytic uremic syndrome involves the concurrence of different susceptibility alleles in the regulators of complement activation gene cluster in 1q32. Human molecular genetics [Internet]. 2005 [cited 2018 Aug 16];14:703–12. Available from: http://www.ncbi.nlm.nih.gov/pubmed/15661753

56. Matar D, Naqvi F, Racusen LC, Carter-Monroe N, Montgomery RA, Alachkar N. Atypical hemolytic uremic syndrome recurrence after kidney transplantation. Transplantation [Internet]. Lippincott Williams and Wilkins; 2014 [cited 2021 Jul 1];98:1205–12. Available from: https://pubmed.ncbi.nlm.nih.gov/24933457/

57. Monteferrante G, Brioschi S, Caprioli J, Pianetti G, Bettinaglio P, Bresin E, et al. Genetic analysis of the complement factor H related 5 gene in haemolytic uraemic syndrome. Molecular Immunology [Internet]. Elsevier Ltd; 2007 [cited 2021 Jul 1];44:1704–8. Available from: https://pubmed.ncbi.nlm.nih.gov/17000000/

58. Tao J, Lieberman J, Lafayette RA, Kambham N. A rare case of Alport syndrome, atypical hemolytic uremic syndrome and Pauci-immune crescentic glomerulonephritis. BMC Nephrology [Internet]. BioMed Central Ltd.; 2018 [cited 2021 Jul 1];19. Available from: https://pubmed.ncbi.nlm.nih.gov/30541482/

59. Payette A, Patey N, Dragon-Durey MA, Frémeaux-Bacchi V, le Deist F, Lapeyraque AL. A case of C3 glomerulonephritis successfully treated with eculizumab. Pediatric Nephrology [Internet]. Springer Verlag; 2015 [cited 2021 Jul 1];30:1033–7. Available from: https://pubmed.ncbi.nlm.nih.gov/25796589/

60. Leroy V, Fremeaux-Bacchi V, Peuchmaur M, Baudouin V, Deschênes G, MacHer MA, et al. Membranoproliferative glomerulonephritis with C3NeF and genetic complement dysregulation. Pediatric Nephrology [Internet]. Pediatr Nephrol; 2011 [cited 2021 Jul 1];26:419–24. Available from: https://pubmed.ncbi.nlm.nih.gov/21188423/

61. Bienaime F, Dragon-Durey MA, Regnier CH, Nilsson SC, Kwan WH, Blouin J, et al. Mutations in components of complement influence the outcome of Factor I-associated atypical hemolytic uremic syndrome. Kidney International [Internet]. Kidney Int; 2010 [cited 2021 Jul 1];77:339–49. Available from: https://pubmed.ncbi.nlm.nih.gov/20016463/

62. Sellier-Leclerc AL, Fremeaux-Bacchi V, Dragon-Durey MA, Macher MA, Niaudet P, Guest G, et al. Differential impact of complement mutations on clinical characteristics in atypical hemolytic uremic syndrome. Journal of the American Society of Nephrology [Internet]. J Am Soc Nephrol; 2007 [cited 2021 Jul 1];18:2392–400. Available from: https://pubmed.ncbi.nlm.nih.gov/17599974/

63. Cayci FS, Cakar N, Hancer VS, Uncu N, Acar B, Gur G. Eculizumab therapy in a child with hemolytic uremic syndrome and CFI mutation. Pediatric Nephrology [Internet]. Pediatr Nephrol; 2012 [cited 2021 Jul 1];27:2327–31. Available from: https://pubmed.ncbi.nlm.nih.gov/22903728/

64. Seddon JM, Yu Y, Miller EC, Reynolds R, Tan PL, Gowrisankar S, et al. Rare variants in CFI, C3 and C9 are associated with high risk of advanced age-related macular degeneration. Nature Genetics. 2013. p. 1366–73.

65. Gleeson PJ, Wilson V, Cox TE, Sharma SD, Smith-Jackson K, Strain L, et al. Chromosomal rearrangement—A rare cause of complement factor I associated atypical haemolytic uraemic syndrome. Immunobiology [Internet]. Elsevier GmbH; 2016 [cited 2021 Jul 1];221:1124–30. Available from: https://pubmed.ncbi.nlm.nih.gov/27268256/

66. Jlajla H, Dehman F, Jallouli M, Khedher R, Ayadi I, Zerzeri Y, et al. Molecular basis of complement factor I deficiency in Tunisian atypical haemolytic and uraemic syndrome patients. Nephrology. Blackwell Publishing; 2019;24:357–64.

67. Nilsson SC, Kalchishkova N, Trouw LA, Fremeaux-Bacchi V, Villoutreix BO, Blom AM. Mutations in complement factor I as found in atypical hemolytic uremic syndrome lead to either altered secretion or altered function of factor I. European Journal of Immunology. 2010;40:172–85.

68. Bluteau O, Sebert M, Leblanc T, Peffault de Latour R, Quentin S, Lainey E, et al. A landscape of germ line mutations in a cohort of inherited bone marrow failure patients. Blood [Internet]. American Society of Hematology; 2018 [cited 2020 Apr 28];131:717–32. Available from: http://www.ncbi.nlm.nih.gov/pubmed/29146883

69. Sethi S, Fervenza FC, Zhang Y, Zand L, Vrana JA, Nasr SH, et al. C3 glomerulonephritis: Clinicopathological findings, complement abnormalities, glomerular proteomic profile, treatment, and follow-up. Kidney International. Nature Publishing Group; 2012;82:465–73.

70. Idorn T, Schejbel L, Rydahl C, Heaf JG, Jølvig KR, Bergstrøm M, et al. Anti-glomerular basement membrane glomerulonephritis and thrombotic microangiopathy in first degree relatives; A case report. BMC Nephrology. 2012;13.

71. Terlizzi V, Castaldo G, Salvatore D, Lucarelli M, Raia V, Angioni A, et al. Genotype-phenotype correlation and functional studies in patients with cystic fibrosis bearing CFTR complex alleles. Journal of Medical Genetics. BMJ Publishing Group; 2017;54:224–35.

72. Claustres M, Altiéri JP, Guittard C, Templin C, Chevalier-Porst F, des Georges M. Are p.1148T, p.R74W and p.D1270N cystic fibrosis causing mutations? BMC Medical Genetics. 2004;5.

73. Brugnon F, Bilan F, Heraud MC, Grizard G, Janny L, Creveaux I. Outcome of intracytoplasmic sperm injection for a couple in which the man is carrier of CFTR p.[R74W;V201M;D1270N] and p.P841R mutations and his spouse a heterozygous carrier of p.F508del mutation of the cystic fibrosis transmembrane conductance regulator gene. Fertility and Sterility. Elsevier Inc.; 2008;90:2004.e23-2004.e26.

74. van Goor F, Yu H, Burton B, Hoffman BJ. Effect of ivacaftor on CFTR forms with missense mutations associated with defects in protein processing or function. Journal of Cystic Fibrosis. 2014;13:29–36.

75. Claustres M, Laussel M, Desgeorges M, Giansily M, Culard JF, Razakatsara G, et al. Analysis of the 27 exons and flanking regions of the cystic fibrosis gene: 40 different mutations account for 91.2% of the mutant alleles in southern france. Human Molecular Genetics. 1993;2:1209–13.

76. Schrijver I, Oitmaa E, Metspalu A, Gardner P. Genotyping microarray for the detection of more than 200 CFTR mutations in ethnically diverse populations. Journal of Molecular Diagnostics. Association of Molecular Pathology; 2005;7:375–87.

77. Tabor HK, Auer PL, Jamal SM, Chong JX, Yu JH, Gordon AS, et al. Pathogenic variants for mendelian and complex traits in exomes of 6,517 european and african americans: Implications for the return of incidental results. American Journal of Human Genetics. Cell Press; 2014;95:183–93.

78. LaRusch J, Jung J, General IJ, Lewis MD, Park HW, Brand RE, et al. Mechanisms of CFTR Functional Variants That Impair Regulated Bicarbonate Permeation and Increase Risk for Pancreatitis but Not for Cystic Fibrosis. PLoS Genetics. Public Library of Science; 2014;10.

79. Castellani C, Cuppens H, Macek M, Cassiman JJ, Kerem E, Durie P, et al. Consensus on the use and interpretation of cystic fibrosis mutation analysis in clinical practice. Journal of Cystic Fibrosis. 2008. p. 179–96.

80. Masica DL, Sosnay PR, Raraigh KS, Cutting GR, Karchin R. Missense variants in CFTR nucleotide-binding domains predict quantitative phenotypes associated with cystic fibrosis disease severity. Human Molecular Genetics. Oxford University Press; 2014;24:1908–17.

81. Sultan M, Werlin S, Venkatasubramani N. Genetic prevalence and characteristics in children with recurrent pancreatitis. Journal of Pediatric Gastroenterology and Nutrition. 2012. p. 645–50.

82. Gallati S, Hess S, Galié-Wunder D, Berger-Menz E, Böhlen D. Cystic fibrosis transmembrane conductance regulator mutations in azoospermic and oligospermic men and their partners. Reproductive biomedicine online [Internet]. Elsevier Ltd; 2009 [cited 2021 Jul 1];19:685–94. Available from: http://www.ncbi.nlm.nih.gov/pubmed/20021716

83. Grangeia A, Alves S, Gonçalves L, Gregório I, Santos AC, Barros H, et al. Spectrum of CFTR gene sequence variants in a northern Portugal population. Pulmonology. Elsevier Espana S.L.U; 2018;24:3–9.

84. Tsui L ‐C. Mutations and sequence variations detected in the cystic fibrosis transmembrane conductance regulator (CFTR) gene: A report from the cystic fibrosis genetic analysis consortium. Human Mutation. 1992;1:197–203.

85. Aref-Eshghi E, Rodenhiser DI, Schenkel LC, Lin H, Skinner C, Ainsworth P, et al. Genomic DNA Methylation Signatures Enable Concurrent Diagnosis and Clinical Genetic Variant Classification in Neurodevelopmental Syndromes. American Journal of Human Genetics. Cell Press; 2018;102:156–74.

86. Marcos S, Sarfati J, Leroy C, Fouveaut C, Parent P, Metz C, et al. The prevalence of CHD7 missense versus truncating mutations is higher in patients with Kallmann syndrome than in typical CHARGE patients. Journal of Clinical Endocrinology and Metabolism. Endocrine Society; 2014;99:E2138–43.

87. Jain S, Kim H-G, Lacbawan F, Meliciani I, Wenzel W, Kurth I, et al. Unique phenotype in a patient with CHARGE syndrome. International Journal of Pediatric Endocrinology. Springer Nature; 2011;2011:11.

88. Schwab C, Gabrysch A, Olbrich P, Patiño V, Warnatz K, Wolff D, et al. Phenotype, penetrance, and treatment of 133 cytotoxic T-lymphocyte antigen 4–insufficient subjects. Journal of Allergy and Clinical Immunology. Mosby Inc.; 2018;142:1932–46.

89. Germeshausen M, Deerberg S, Peter Y, Reimer C, Kratz CP, Ballmaier M. The Spectrum of ELANE Mutations and their Implications in Severe Congenital and Cyclic Neutropenia. Human Mutation. 2013;34:905–14.

90. Bellanné-Chantelot C, Clauin S, Leblanc T, Cassinat B, Rodrigues-Lima F, Beaufils S, et al. Mutations in the ELA2 gene correlate with more severe expression of neutropenia: A study of 81 patients from the French Neutropenia Register. Blood. 2004;103:4119–25.

91. Alizadeh Z, Fazlollahi MR, Houshmand M, Maddah M, Chavoshzadeh Z, Hamidieh AA, et al. Different pattern of gene mutations in Iranian patients with severe congenital neutropenia (including 2 new mutations). Iranian Journal of Allergy, Asthma and Immunology. 2013;12:86–92.

92. Xia J, Link DC. Severe congenital neutropenia and the unfolded protein response. Current Opinion in Hematology. 2008. p. 1–7.

93. Horwitz MS, Duan Z, Korkmaz B, Lee HH, Mealiffe ME, Salipante SJ. Neutrophil elastase in cyclic and severe congenital neutropenia [Internet]. Blood. Blood; 2007 [cited 2021 Jul 1]. p. 1817–24. Available from: https://pubmed.ncbi.nlm.nih.gov/17053055/

94. Demuth I, Wlodarski M, Tipping AJ, Morgan N v., de Winter JP, Thiel M, et al. Spectrum of mutations in the Fanconi anaemia group G gene, FANCG/XRCC9. European Journal of Human Genetics. 2000;8:861–8.

95. Nakanishi K, Moran A, Hays T, Kuang Y, Fox E, Garneau D, et al. Functional analysis of patient-derived mutations in the Fanconi anemia gene, FANCG/XRCC9. Experimental Hematology. 2001. p. 842–9.

96. Boggio E, Aricò M, Melensi M, Dianzani I, Ramenghi U, Dianzani U, et al. Mutation of FAS, XIAP, and UNC13D genes in a patient with a complex lymphoproliferative phenotype. Pediatrics. 2013;132.

97. Campagnoli MF, Garbarini L, Quarello P, Garelli E, Carando A, Baravalle V, et al. The broad spectrum of autoimmune lymphoproliferative disease: Molecular bases, clinical features and long-term follow-up in 31 patients. Haematologica. 2006;91:538–41.

98. Wada T, Yasui M, Toma T, Nakayama Y, Nishida M, Shimizu M, et al. Detection of T lymphocytes with a second-site mutation in skin lesions of atypical X-linked severe combined immunodeficiency mimicking Omenn syndrome. Blood [Internet]. Blood; 2008 [cited 2021 Jul 1];112:1872–5. Available from: https://pubmed.ncbi.nlm.nih.gov/18559672/

99. Mørk N, Kofod-Olsen E, Sørensen KB, Bach E, Ørntoft TF, Østergaard L, et al. Mutations in the TLR3 signaling pathway and beyond in adult patients with herpes simplex encephalitis. Genes and Immunity [Internet]. Nature Publishing Group; 2015 [cited 2021 Jul 1];16:552–66. Available from: https://pubmed.ncbi.nlm.nih.gov/26513235/

100. Baer S, Afenjar A, Smol T, Piton A, Gérard B, Alembik Y, et al. Wiedemann-Steiner syndrome as a major cause of syndromic intellectual disability: A study of 33 French cases. Clinical Genetics [Internet]. Blackwell Publishing Ltd; 2018 [cited 2021 Jul 1];94:141–52. Available from: https://pubmed.ncbi.nlm.nih.gov/29574747/

101. Banka S, Veeramachaneni R, Reardon W, Howard E, Bunstone S, Ragge N, et al. How genetically heterogeneous is Kabuki syndrome: MLL2 testing in 116 patients, review and analyses of mutation and phenotypic spectrum. European Journal of Human Genetics. 2012. p. 381–8.

102. Faundes V, Malone G, Newman WG, Banka S. A comparative analysis of KMT2D missense variants in Kabuki syndrome, cancers and the general population. Journal of Human Genetics. Nature Publishing Group; 2019;64:161–70.

103. Micale L, Augello B, Maffeo C, Selicorni A, Zucchetti F, Fusco C, et al. Molecular Analysis, Pathogenic Mechanisms, and Readthrough Therapy on a Large Cohort of Kabuki Syndrome Patients. Human Mutation. John Wiley and Sons Inc.; 2014;35:841–50.

104. Altug U, Ensari C, Sayin DB, Ensari A. MEFV gene mutations in Henoch-Schönlein purpura. International journal of rheumatic diseases. 2013;16:347–51.

105. Arai Y, Yamashita K, Mizugishi K, Watanabe T, Kondo T, Kitano T, et al. Familial Mediterranean fever mutations in a patient with recurrent episodes of acute respiratory distress syndrome. Clinical Immunology. 2013. p. 58–60.

106. Moradian MM, Sarkisian T, Amaryan G, Hayrapetyan H, Yeghiazaryan A, Davidian N, et al. Patient management and the association of less common familial Mediterranean fever symptoms with other disorders. Genetics in Medicine. Lippincott Williams and Wilkins; 2014;16:258–63.

107. Feng J, Zhang Z, Li W, Shen X, Song W, Yang C, et al. Missense mutations in the MEFV gene are associated with fibromyalgia syndrome and correlate with elevated IL-1β plasma levels. PLoS ONE. 2009;4.

108. Aksentijevich I, Torosyan Y, Samuels J, Centola M, Pras E, Chae JJ, et al. Mutation and haplotype studies of familial Mediterranean fever reveal new ancestral relationships and evidence for a high carrier frequency with reduced penetrance in the Ashkenazi Jewish population. American Journal of Human Genetics. University of Chicago Press; 1999;64:949–62.

109. Cazeneuve C, Sarkisian T, Pêcheux C, Dervichian M, Nédelec B, Reinert P, et al. MEFV-gene analysis in Armenian patients with familial Mediterranean fever: Diagnostic value and unfavorable renal prognosis of the M694V homozygous genotype - Genetic and therapeutic implications. American Journal of Human Genetics. University of Chicago Press; 1999;65:88–97.

110. Joo K, Park W, Chung MH, Lim MJ, Jung KH, Heo Y, et al. Extensive thrombosis in a patient with familial mediterranean fever, despite hyperimmunoglobulin D state in serum - first adult case in Korea. Journal of Korean Medical Science. 2013;28:328–30.

111. Migita K, Agematsu K, Yazaki M, Nonaka F, Nakamura A, Toma T, et al. Familial mediterranean fever: Genotype-phenotype correlations in japanese patients. Medicine (United States). Lippincott Williams and Wilkins; 2014;93:158–64.

112. Yamagami K, Nakamura T, Nakamura R, Hanioka Y, Seki K, Chiba H, et al. Familial Mediterranean fever with P369S/R408Q exon3 variant in pyrin presenting as symptoms of PFAPA. Modern Rheumatology. Taylor and Francis Ltd; 2017;27:356–9.

113. Aldea A, Casademont J, Aróstegui JI, Rius J, Masó M, Vives J, et al. I591T MEFV mutation in a Spanish kindred: Is it a mild mutation, a benign polymorphism, or a variant influenced by another modifier? Human Mutation. 2002. p. 148–50.

114. Stella A, Cortellessa F, Scaccianoce G, Pivetta B, Settimo E, Portincasa P. Familial Mediterranean fever: Breaking all the (genetic) rules. Rheumatology (United Kingdom). Oxford University Press; 2019;58:463–7.

115. Touitou I. The spectrum of Familial Mediterranean Fever (FMF) mutations. European Journal of Human Genetics. 2001. p. 473–83.

116. Šedivá A, Horváth R, Maňásek V, Gregorová A, Plevová P, Horáčková M, et al. Cluster of patients with Familial Mediterranean fever and heterozygous carriers of mutations in MEFV gene in the Czech Republic. Clinical Genetics. 2014;86:564–9.

117. Bernot A, da Silva C, Petit JL, Cruaud C, Caloustian C, Castet V, et al. Non-founder mutations in the MEFV gene establish this gene as the cause of familial Mediterranean fever (FMF). Human Molecular Genetics. Oxford University Press; 1998;7:1317–25.

118. Mansour I, Delague V, Cazeneuve C, Dodé C, Chouery E, Pêcheux C, et al. Familial Mediterranean fever in Lebanon: Mutation spectrum, evidence for cases in Maronites, Greek orthodoxes, Greek catholics, Syriacs and Chiites and for an association between amyloidosis and M694V and M694l mutations. European Journal of Human Genetics. Nature Publishing Group; 2001;9:51–5.

119. Chalevelakis G, Apostolakis I, Koliou X, Apessos A, Kyriakopoulou V, Vrakidou E, et al. Different intrafamilial clinical presentation of FMF mutation carriers. Genetic Testing. 2008;12:125–7.

120. Lainka E, Bielak M, Lohse P, Timmann C, Stojanov S, von Kries R, et al. Familial Mediterranean fever in Germany: Epidemiological, clinical, and genetic characteristics of a pediatric population. European Journal of Pediatrics. 2012;171:1775–85.

121. Kriegshäuser G, Enko D, Hayrapetyan H, Atoyan S, Oberkanins C, Sarkisian T. Clinical and genetic heterogeneity in a large cohort of Armenian patients with late-onset familial Mediterranean fever. Genetics in Medicine. Nature Publishing Group; 2018;20:1583–8.

122. Kilinc M, Ganiyusufoglu E, Sager H, Celik A, Olgar S, Cetin GY, et al. The report of sequence analysis on familial Mediterranean fever gene (MEFV) in South-eastern Mediterranean region (Kahramanmaraş) of Turkey. Rheumatology International [Internet]. Springer Verlag; 2016 [cited 2021 Jul 1];36:25–31. Available from: https://pubmed.ncbi.nlm.nih.gov/26215181/

123. Domingo C, Touitou I, Bayou A, Ozen S, Notarnicola C, Dewalle M, et al. Familial Mediterranean fever in the “Chuetas” of Mallorca: A question of Jewish origin or genetic heterogeneity. European Journal of Human Genetics. 2000;8:242–6.

124. Oshima K, Yamazaki K, Nakajima Y, Kobayashi A, Kato T, Ohara O, et al. A case of familial Mediterranean fever associated with compound heterozygosity for the pyrin variant L110P-E148Q/M680I in Japan. Modern Rheumatology. 2010;20:193–5.

125. Inoue K, Torii K, Yoda A, Kadota K, Nakamichi S, Obata Y, et al. Familial mediterranean fever with onset at 66 years of age. Internal Medicine. 2012;51:2649–53.

126. Lim AL, Jang HJ, Han JW, Song YK, Song WJ, Woo HJ, et al. Familial Mediterranean fever: The first adult case in Korea. Journal of Korean Medical Science. 2012;27:1424–7.

127. Eguchi M, Miyashita T, Shirouzu H, Sato S, Izumi Y, Takeoka A, et al. Coexistence of polymyositis and familial Mediterranean fever. Modern Rheumatology. 2013;23:374–8.

128. Ashida M, Koike Y, Kuwatsuka S, Ichinose K, Migita K, Sano S, et al. Psoriasis-like lesions in a patient with familial Mediterranean fever. Journal of Dermatology. Blackwell Publishing Ltd; 2016;43:314–7.

129. Cantarini L, Lucherini OM, Simonini G, Galeazzi M, Baldari CT, Cimaz R. Systemic-onset juvenile idiopathic arthritis complicated by early onset amyloidosis in a patient carrying a mutation in the MEFV gene. Rheumatology International [Internet]. Rheumatol Int; 2012 [cited 2021 Jul 1];32:465–7. Available from: https://pubmed.ncbi.nlm.nih.gov/20044784/

130. Jéru I, Hentgen V, Normand S, Duquesnoy P, Cochet E, Delwail A, et al. Role of interleukin-1β in NLRP12-associated autoinflammatory disorders and resistance to anti-interleukin-1 therapy. Arthritis and Rheumatism. 2011;63:2142–8.

131. Jéru I, Duquesnoy P, Fernandes-Alnemri T, Cochet E, Yu JW, Lackmy-Port-Lis M, et al. Mutations in NALP12 cause hereditary periodic fever syndromes. Proceedings of the National Academy of Sciences of the United States of America. 2008;105:1614–9.

132. Ledesma PA, Guerra JC, Burbano M, Procel P, Pedroza LA. Whole exome sequencing in a child with acute disseminated encephalomyelitis, optic neuritis, and periodic fever syndrome: A case report. Journal of Medical Case Reports. BioMed Central Ltd.; 2019;13.

133. Rusmini M, Federici S, Caroli F, Grossi A, Baldi M, Obici L, et al. Next-generation sequencing and its initial applications for molecular diagnosis of systemic auto-inflammatory diseases. Annals of the Rheumatic Diseases. BMJ Publishing Group; 2016;75:1550–7.

134. Kostik MM, Suspitsin EN, Guseva MN, Levina AS, Kazantseva AY, Sokolenko AP, et al. Multigene sequencing reveals heterogeneity of NLRP12-related autoinflammatory disorders. Rheumatology International. Springer Verlag; 2018;38:887–93.

135. Borte S, Celiksoy MH, Menzel V, Ozkaya O, Ozen FZ, Hammarström L, et al. Novel NLRP12 mutations associated with intestinal amyloidosis in a patient diagnosed with common variable immunodeficiency. Clinical Immunology. Academic Press Inc.; 2014;154:105–11.

136. Verma D, Särndahl E, Andersson H, Eriksson P, Fredrikson M, Jönsson J-I, et al. The Q705K polymorphism in NLRP3 is a gain-of-function alteration leading to excessive interleukin-1β and IL-18 production. PloS one. 2012;7:e34977.

137. Vitale A, Lucherini O, Galeazzi M, Frediani B, Cantarini L. Long-term clinical course of patients carrying the Q703K mutation in the NLRP3 gene: a case series. Clin Exp Rheumatol. 2012;30:943–6.

138. Rieber N, Gavrilov A, Hofer L, Singh A, Öz H, Endres T, et al. A functional inflammasome activation assay differentiates patients with pathogenic NLRP3 mutations and symptomatic patients with low penetrance variants. Clinical Immunology [Internet]. 2015 [cited 2017 Oct 9];157:56–64. Available from: http://www.ncbi.nlm.nih.gov/pubmed/25596455

139. Verma D, Lerm M, Julinder RB, Eriksson P, Söderkvist P, Särndahl E. Gene polymorphisms in the NALP3 inflammasome are associated with interleukin-1 production and severe inflammation relation to common inflammatory diseases? Arthritis and Rheumatism. 2008;58:888–94.

140. Ting T v., Scalzi L v., Hashkes PJ. Nonclassic Neurologic Features in Cryopyrin-Associated Periodic Syndromes. Pediatric Neurology. 2007;36:338–41.

141. Perko D, Debeljak M, Toplak N, Avčin T. Clinical features and genetic background of the periodic fever syndrome with aphthous stomatitis, pharyngitis, and adenitis: A single center longitudinal study of 81 patients. Mediators of Inflammation [Internet]. Hindawi Publishing Corporation; 2015 [cited 2021 Jul 1];2015. Available from: https://pubmed.ncbi.nlm.nih.gov/25821352/

142. Jesus AA, Fujihira E, Watase M, Terreri MT, Hilario MO, Carneiro-Sampaio M, et al. Hereditary autoinflammatory syndromes: A Brazilian multicenter study. Journal of Clinical Immunology. Springer New York LLC; 2012;32:922–32.

143. Yüksel Ş, Eren E, Hatemi G, Sahillioǧlu AC, Gültekin Y, DemirÖz D, et al. Novel NLRP3/cryopyrin mutations and pro-inflammatory cytokine profiles in behçet’s syndrome patients. International Immunology. 2014;26:71–81.

144. Kuemmerle-Deschner JB, Koitschev A, Tyrrell PN, Plontke SK, Deschner N, Hansmann S, et al. Early detection of sensorineural hearing loss in Muckle-Wells-syndrome. Pediatric Rheumatology. BioMed Central Ltd.; 2015;13.

145. Rowczenio DM, Trojer H, Russell T, Baginska A, Lane T, Stewart NM, et al. Clinical characteristics in subjects with NLRP3 V198M diagnosed at a single UK center and a review of the literature. Arthritis Research and Therapy. 2013;15.

146. Hoffman HM, Mueller JL, Broide DH, Wanderer AA, Kolodner RD. Mutation of a new gene encoding a putative pyrin-like protein causes familial cold autoinflammatory syndrome and Muckle-Wells syndrome. Nature Genetics. 2001;29:301–5.

147. Rosé CD, Pans S, Casteels I, Anton J, Bader-Meunier B, Brissaud P, et al. Blau syndrome: Cross-sectional data from a multicentre study of clinical, radiological and functional outcomes. Rheumatology (United Kingdom) [Internet]. Oxford University Press; 2015 [cited 2021 Jul 1];54:1008–16. Available from: https://pubmed.ncbi.nlm.nih.gov/25416713/

148. Ogunjimi B, Zhang SY, Sorensen KB, Skipper KA, Carter-Timofte M, Kerner G, et al. Inborn errors in RNA polymerase III underlie severe varicella zoster virus infections. Journal of Clinical Investigation [Internet]. American Society for Clinical Investigation; 2017 [cited 2021 Jul 1];127:3543–56. Available from: https://pubmed.ncbi.nlm.nih.gov/28783042/

149. Solomou EE, Gibellini F, Stewart B, Malide D, Berg M, Visconte V, et al. Perforin gene mutations in patients with acquired aplastic anemia. Blood [Internet]. 2007 [cited 2018 Oct 29];109:5234–7. Available from: http://www.ncbi.nlm.nih.gov/pubmed/17311987

150. Starnes TW, Bennin DA, Bing X, Eickhoff JC, Grahf DC, Bellak JM, et al. The F-BAR protein PSTPIP1 controls extracellular matrix degradation and filopodia formation in macrophages. Blood. American Society of Hematology; 2014;123:2703–14.

151. Calderón-Castrat X, Bancalari-Díaz D, Román-Curto C, Romo-Melgar A, Amorós-Cerdán D, Alcaraz-Mas LA, et al. PSTPIP1 gene mutation in a pyoderma gangrenosum, acne and suppurative hidradenitis (PASH) syndrome. British Journal of Dermatology. Blackwell Publishing Ltd; 2016. p. 194–8.

152. Depner M, Fuchs S, Raabe J, Frede N, Glocker C, Doffinger R, et al. The Extended Clinical Phenotype of 26 Patients with Chronic Mucocutaneous Candidiasis due to Gain-of-Function Mutations in STAT1. Journal of Clinical Immunology. Springer New York LLC; 2016;36:73–84.

153. Uzel G, Sampaio EP, Lawrence MG, Hsu AP, Hackett M, Dorsey MJ, et al. Dominant gain-of-function STAT1 mutations in FOXP3 wild-type immune dysregulation-polyendocrinopathy-enteropathy-X-linked-like syndrome. Journal of Allergy and Clinical Immunology. Mosby Inc.; 2013;131.

154. Szymanski EP, Leung JM, Fowler CJ, Haney C, Hsu AP, Chen F, et al. Pulmonary nontuberculous mycobacterial infection a multisystem, multigenic disease. American Journal of Respiratory and Critical Care Medicine. American Thoracic Society; 2015;192:618–28.

155. van Schouwenburg PA, Davenport EE, Kienzler AK, Marwah I, Wright B, Lucas M, et al. Application of whole genome and RNA sequencing to investigate the genomic landscape of common variable immunodeficiency disorders. Clinical Immunology. 2015;

156. Yamaguchi H, Calado RT, Ly H, Kajigaya S, Baerlocher GM, Chanock SJ, et al. Mutations in TERT, the Gene for Telomerase Reverse Transcriptase, in Aplastic Anemia . New England Journal of Medicine. Massachusetts Medical Society; 2005;352:1413–24.

157. Du HY, Pumbo E, Manley P, Field JJ, Bayliss SJ, Wilson DB, et al. Complex inheritance pattern of dyskeratosis congenita in two families with 2 different mutations in the telomerase reverse transcriptase gene. Blood. American Society of Hematology; 2008;111:1128–30.

158. Calado RT, Regal JA, Kajigaya S, Young NS. Erosion of telomeric single-stranded overhang in patients with aplastic anaemia carrying telomerase complex mutations. European Journal of Clinical Investigation. 2009;39:1025–32.

159. Zaug AJ, Crary SM, Jesse Fioravanti M, Campbell K, Cech TR. Many disease-associated variants of hTERT retain high telomerase enzymatic activity. Nucleic Acids Research. 2013;41:8969–78.

160. Gutierrez-Rodrigues F, Donaires FS, Pinto A, Vicente A, Dillon LW, Clé D v., et al. Pathogenic TERT promoter variants in telomere diseases. Genetics in Medicine. Nature Publishing Group; 2019;21:1594–602.

161. Gramatges MM, Qi X, Sasa GS, Chen JJL, Bertuch AA. A homozygous telomerase T-motif variant resulting in markedly reduced repeat addition processivity in siblings with Hoyeraal Hreidarsson syndrome. Blood. American Society of Hematology; 2013;121:3586–93.

162. Matsumoto T, Fan X, Ishikawa E, Ito M, Amano K, Toyoda H, et al. Analysis of patients with atypical hemolytic uremic syndrome treated at the Mie University Hospital: Concentration of C3 p.I1157T mutation. International Journal of Hematology. Springer Japan; 2014;100:437–42.

163. Zhao W, Ding Y, Lu J, Zhang T, Chen D, Zhang H, et al. Genetic analysis of the complement pathway in C3 glomerulopathy. Nephrology Dialysis Transplantation. Oxford University Press; 2018;33:1919–27.

164. Delvaeye M, Noris M, de Vriese A, Esmon CT, Esmon NL, Ferrell G, et al. Thrombomodulin mutations in atypical hemolytic-uremic syndrome. The New England journal of medicine [Internet]. 2009;361:345–57. Available from: http://www.ncbi.nlm.nih.gov/pubmed/19625716

165. Noris M, Caprioli J, Bresin E, Mossali C, Pianetti G, Gamba S, et al. Relative Role of Genetic Complement Abnormalities in Sporadic and Familial aHUS and Their Impact on Clinical Phenotype. Clinical Journal of the American Society of Nephrology. 2010;5:1844–59.

166. Caroti L, di Maria L, Carta P, Moscarelli L, Cirami C, Minetti EE. Posttransplant outcome of atypical haemolytic uraemic syndrome in a patient with thrombomodulin mutation: a case without recurrence. Clinical kidney journal [Internet]. Oxford University Press; 2015 [cited 2021 Jul 1];8:329–31. Available from: http://www.ncbi.nlm.nih.gov/pubmed/26034596

167. Hoffman TW, van der Vis JJ, van Oosterhout MFM, van Es HW, van Kessel DA, Grutters JC, et al. TINF2 Gene Mutation in a Patient with Pulmonary Fibrosis. Case reports in pulmonology [Internet]. Hindawi Limited; 2016 [cited 2021 Jul 1];2016:1310862. Available from: http://www.ncbi.nlm.nih.gov/pubmed/27088026

168. Walne AJ, Vulliamy T, Beswick R, Kirwan M, Dokal I. TINF2 mutations result in very short telomeres: Analysis of a large cohort of patients with dyskeratosis congenita and related bone marrow failure syndromes. Blood. 2008;112:3594–600.

169. Salzer U, Bacchelli C, Buckridge S, Pan-Hammarström Q, Jennings S, Lougaris V, et al. Relevance of biallelic versus monoallelic TNFRSF13B mutations in distinguishing disease-causing from risk-increasing TNFRSF13B variants in antibody deficiency syndromes. Blood. 2009;113:1967–76.

170. Lougaris V, Gallizzi R, Vitali M, Baronio M, Salpietro A, Bergbreiter A, et al. A novel compound heterozygous TACI mutation in an autosomal recessive common variable immunodeficiency (CVID) family. Human Immunology. 2012;73:836–9.

171. Freiberger T, Ravčuková B, Grodecká L, Pikulová Z, Štikarovská D, PeŠák S, et al. Sequence variants of the TNFRSF13B gene in Czech CVID and IgAD patients in the context of other populations. Human Immunology. 2012;73:1147–54.

172. Chi ZH, Wei W, Bu DF, Li HH, Ding F, Zhu P. Targeted high-throughput sequencing technique for the molecular diagnosis of primary immunodeficiency disorders. Medicine (United States). Lippincott Williams and Wilkins; 2018;97.

173. Salzer U, Chapel HM, Webster ADB, Pan-Hammarström Q, Schmitt-Graeff A, Schlesier M, et al. Mutations in TNFRSF13B encoding TACI are associated with common variable immunodeficiency in humans. Nature Genetics. 2005;37:820–8.

174. Berglund LJ, Jones GJ, Murali R, Fulcher DA. TACI mutation with invasive polyclonal CD8 + T-cell lymphoproliferation in a patient with common variable immunodeficiency. Journal of Allergy and Clinical Immunology. 2006;117:870–7.

175. Lee JJ, Jabara HH, Garibyan L, Rauter I, Sannikova T, Dillon SR, et al. The C104R mutant impairs the function of transmembrane activator and calcium modulator and cyclophilin ligand interactor (TACI) through haploinsufficiency. Journal of Allergy and Clinical Immunology. Mosby Inc.; 2010;126.

176. Pulvirenti F, Zuntini R, Milito C, Specchia F, Spadaro G, Danieli MG, et al. Clinical Associations of Biallelic and Monoallelic TNFRSF13B Variants in Italian Primary Antibody Deficiency Syndromes. Journal of Immunology Research. Hindawi Limited; 2016;2016.

177. de Valles-Ibáñez G, Esteve-Solé A, Piquer M, Azucena González-Navarro E, Hernandez-Rodriguez J, Laayouni H, et al. Evaluating the genetics of common variable immunodeficiency: Monogenetic model and beyond. Frontiers in Immunology. Frontiers Media S.A.; 2018;9.

178. Abolhassani H, Aghamohammadi A, Fang M, Rezaei N, Jiang C, Liu X, et al. Clinical implications of systematic phenotyping and exome sequencing in patients with primary antibody deficiency. Genetics in Medicine. Nature Publishing Group; 2019;21:243–51.

179. Dong X, Hoeltzle M v., Hagan JB, Park MA, Li JT, Abraham RS. Phenotypic and clinical heterogeneity associated with monoallelic TNFRSF13B-A181E mutations in common variable immunodeficiency. Human Immunology. 2010;71:505–11.

180. Martinez-Gallo M, Radigan L, Almejún MB, Martínez-Pomar N, Matamoros N, Cunningham-Rundles C. TACI mutations and impaired B-cell function in subjects with CVID and healthy heterozygotes. Journal of Allergy and Clinical Immunology. 2013;131:468–76.

181. Romberg N, Virdee M, Chamberlain N, Oe T, Schickel JN, Perkins T, et al. TNF receptor superfamily member 13b (TNFRSF13B) hemizygosity reveals transmembrane activator and CAML interactor haploinsufficiency at later stages of B-cell development. Journal of Allergy and Clinical Immunology. Mosby Inc.; 2015;136:1315–25.

182. Nedjai B, Hitman GA, Church LD, Minden K, Whiteford ML, McKee S, et al. Differential cytokine secretion results from p65 and c-Rel NF-κB subunit signaling in peripheral blood mononuclear cells of TNF receptor-associated periodic syndrome patients. Cellular Immunology. Academic Press; 2011;268:55–9.

183. Cantarini L, Rigante D, Merlini G, Vitale A, Caso F, Lucherini OM, et al. The expanding spectrum of low-penetrance TNFRSF1A gene variants in adults presenting with recurrent inflammatory attacks: Clinical manifestations and long-term follow-up. Seminars in Arthritis and Rheumatism. W.B. Saunders; 2014;43:818–23.

184. Ravet N, Rouaghe S, Dodé C, Bienvenu J, Stirnemann J, Lévy P, et al. Clinical significance of P46L and R92Q substitutions in the tumour necrosis factor superfamily 1A gene. Annals of the Rheumatic Diseases. 2006;65:1158–62.

185. Aksentijevich I, Galon J, Soares M, Mansfield E, Hull K, Oh HH, et al. The tumor-necrosis-factor receptor-associated periodic syndrome: New mutations in TNFRSF1A, ancestral origins, genotype-phenotype studies, and evidence for further genetic heterogeneity of periodic fevers. American Journal of Human Genetics. 2001;69:301–14.

186. Hoffmann F, Lohse P, Stojanov S, Shin YS, Renner ED, Kéry A, et al. Identification of a novel mevalonate kinase gene mutation in combination with the common MVK V3771 substitution and the low-penetrance TNFRSF1A R92Q mutation. European Journal of Human Genetics. 2005;13:510–2.

187. Hoang TK, Albert DA. Novel presentations of periodic fever syndromes: Discrepancies between genetic and clinical diagnoses. European Journal of Rheumatology. AVES Publishing Co.; 2019;6:12–8.

188. Ruiz-Ortiz E, Iglesias E, Soriano A, Buján-Rivas S, Español-Rego M, Castellanos-Moreira R, et al. Disease phenotype and outcome depending on the age at disease onset in patients carrying the R92Q low-penetrance variant in TNFRSF1A gene. Frontiers in Immunology. Frontiers Research Foundation; 2017;8.

189. Krelenbaum M, Chaiton A. Successful treatment with infliximab of a patient with Tumor Necrosis Factor-associated Periodic Syndrome (TRAPS) who failed to respond to etanercept. Journal of Rheumatology. 2010. p. 1780–2.

190. Bachetti T, Chiesa S, Castagnola P, Bani D, di Zanni E, Omenetti A, et al. Autophagy contributes to inflammation in patients with TNFR-associated periodic syndrome (TRAPS). Annals of the Rheumatic Diseases. 2013;72:1044–52.

191. Li Q, Lee CH, Peters LA, Mastropaolo LA, Thoeni C, Elkadri A, et al. Variants in TRIM22 That Affect NOD2 Signaling Are Associated with Very-Early-Onset Inflammatory Bowel Disease. Gastroenterology [Internet]. W.B. Saunders; 2016 [cited 2021 Jul 1];150:1196–207. Available from: https://pubmed.ncbi.nlm.nih.gov/26836588/

192. Aricò M, Boggio E, Cetica V, Melensi M, Orilieri E, Clemente N, et al. Variations of the UNC13D Gene in Patients with Autoimmune Lymphoproliferative Syndrome. PLoS ONE. 2013;8:1–9.

193. Santoro A, Cannella S, Bossi G, Gallo F, Trizzino A, Pende D, et al. Novel Munc13-4 mutations in children and young adult patients with haemophagocytic lymphohistiocytosis. Journal of medical genetics [Internet]. 2006 [cited 2017 Nov 16];43:953–60. Available from: http://jmg.bmj.com/cgi/doi/10.1136/jmg.2006.041863

194. Zhang K, Jordan MB, Marsh RA, Johnson JA, Kissell D, Meller J, et al. Hypomorphic mutations in PRF1, MUNC13-4, and STXBP2 are associated with adult-onset familial HLH. Blood. 2011;118:5794–8.

195. Jin Z, Wang Y, Wang J, Zhang J, Wu L, Gao Z, et al. Primary hemophagocytic lymphohistiocytosis in adults: the utility of family surveys in a single-center study from China. Orphanet Journal of Rare Diseases [Internet]. 2018 [cited 2018 Oct 29];13:17. Available from: http://www.ncbi.nlm.nih.gov/pubmed/29357941

196. Chen X, Zhang Y, Wang F, Wang M, Teng W, Lin Y, et al. Germline cytotoxic lymphocytes defective mutations in Chinese patients with lymphoma. Oncology Letters. Spandidos Publications; 2017;14:5249–56.

197. Xu XJ, Wang HS, Ju XL, Xiao PF, Xiao Y, Xue HM, et al. Clinical presentation and outcome of pediatric patients with hemophagocytic lymphohistiocytosis in China: A retrospective multicenter study. Pediatric Blood and Cancer. John Wiley and Sons Inc.; 2017;64.

198. Chen X, Wang F, Zhang Y, Teng W, Wang M, Nie D, et al. Genetic variant spectrum in 265 Chinese patients with hemophagocytic lymphohistiocytosis: Molecular analyses of PRF1, UNC13D, STX11, STXBP2, SH2D1A, and XIAP. Clinical Genetics [Internet]. Blackwell Publishing Ltd; 2018 [cited 2021 Jul 1];94:200–12. Available from: https://pubmed.ncbi.nlm.nih.gov/29665027/
