# Supplementary material for: Prevalence of Pathogenic and Potentially Pathogenic Inborn Error of Immunity Associated Variants in Children with Severe Sepsis
Source: J Clin Immunol. 2022 Jan 1;42(2):350–64. doi: 10.1007/s10875-021-01183-4 (PMC8720168; doi:10.1007/s10875-021-01183-4)
Supplement: Supplementary file 5 — Supplementary file5 (PDF 49 KB) [file 10875_2021_1183_MOESM5_ESM.pdf]

	Culture Negative				Blood Culture				Urine Culture				Respiratory Culture				Respiratory Virus			
	OR	95% CI	Fishers *p* value	BH adjusted *p* value	OR	95% CI	Fishers *p* value	BH adjusted *p* value	OR	95% CI	Fishers *p* value	BH adjusted *p* value	OR	95% CI	Fishers *p* value	BH adjusted *p* value	OR	95% CI	Fishers *p* value	BH adjusted *p* value
Any	0.6	(0.4 - 0.9)	0.0131	0.0721	2.8	(1.1 - 7.1)	0.030	0.082	8.2	(1.1 - 64.1)	0.0186	0.0683	1.6	(0.9 - 2.7)	0.0897	0.2358	1.6	(0.9 - 2.8)	0.1062	0.5794
Multiple	0.4	(0.2 - 0.7)	0.0042	0.0463	4.3	(1.5 - 12.5)	0.008	0.046	9.6	(1.0 - 87.5)	0.0322	0.0709	2.2	(1.1 - 4.4)	0.0275	0.1513	1.3	(0.6 - 2.9)	0.5474	0.7527
Complement	0.6	(0.3 - 1.0)	0.0411	0.1123	2.8	(1.0 - 7.9)	0.070	0.154	7.4	(0.9 - 64.6)	0.0842	0.1322	1.7	(0.9 - 3.3)	0.1072	0.2358	1.3	(0.7 - 2.5)	0.4927	0.7527
Autoinflammation	0.6	(0.3 - 1.0)	0.0612	0.1123	2.4	(0.8 - 7.2)	0.151	0.238	9.0	(1.0 - 78.2)	0.0276	0.0709	1.6	(0.8 - 3.1)	0.2258	0.3549	1.4	(0.7 - 2.8)	0.3682	0.7527
Combined Syndromic	0.5	(0.2 - 1.1)	0.1377	0.1894	4.7	(1.3 - 16.7)	0.023	0.082	0.0	(0.0 - NA)	1.0000	1.0000	2.9	(1.2 - 7.0)	0.0229	0.1513	2.3	(0.9 - 5.8)	0.1116	0.5794
Innate	0.6	(0.2 - 1.4)	0.2397	0.2636	6.9	(1.9 - 25.3)	0.007	0.046	6.8	(0.4 - 113.1)	0.2497	0.3433	1.4	(0.5 - 4.2)	0.5534	0.6764	1.6	(0.5 - 4.7)	0.5362	0.7527
Phagocyte	0.3	(0.1 - 1.0)	0.0554	0.1123	1.5	(0.2 - 13.2)	0.542	0.542	32.3	(3.1 - 334.5)	0.0034	0.0377	2.8	(0.9 - 8.6)	0.0913	0.2358	1.2	(0.3 - 4.5)	0.7333	0.8066
Dysregulation	0.4	(0.1 - 1.1)	0.1108	0.1742	3.0	(0.5 - 16.0)	0.213	0.261	0.0	(0.0 - NA)	1.0000	1.0000	1.0	(0.3 - 3.7)	1.0000	1.0000	1.1	(0.3 - 4.1)	1.0000	1.0000
Bone Marrow	1.3	(0.4 - 4.3)	0.7729	0.7729	1.7	(0.2 - 15.5)	0.495	0.542	0.0	(0.0 - NA)	1.0000	1.0000	0.8	(0.2 - 3.7)	1.0000	1.0000	1.4	(0.4 - 5.5)	0.7054	0.8066
Ab	0.4	(0.1 - 1.4)	0.1905	0.2328	5.2	(0.9 - 29.8)	0.102	0.187	32.3	(2.6 - 394.6)	0.0133	0.0683	1.8	(0.4 - 7.5)	0.4185	0.5754	2.0	(0.5 - 8.3)	0.3944	0.7527
Cellular & Humoral	0.0	(0.0 - NA)	0.0464	0.1123	6.9	(0.6 - 76.5)	0.195	0.261	43.0	(2.1 - 862.7)	0.0590	0.1082	4.2	(0.6 - 31.3)	0.1808	0.3314	4.7	(0.6 - 34.8)	0.1580	0.5794
