# Supplementary material for: Prevalence of Pathogenic and Potentially Pathogenic Inborn Error of Immunity Associated Variants in Children with Severe Sepsis
Source: J Clin Immunol. 2022 Jan 1;42(2):350–64. doi: 10.1007/s10875-021-01183-4 (PMC8720168; doi:10.1007/s10875-021-01183-4)
Supplement: Supplementary file 6 — Supplementary file6 (PDF 46 KB) [file 10875_2021_1183_MOESM6_ESM.pdf]

	Ferritin >500ng/ml				CRP >10mg/dl				Plts < 150,000/ul				Lymphocytes <1000/ul			
	OR	95% CI	Fishers *p* value	BH adjusted *p* value	OR	95% CI	Fishers *p* value	BH adjusted *p* value	OR	95% CI	Fishers *p* value	BH adjusted *p* value	OR	95% CI	Fishers *p* value	BH adjusted *p* value
Any	2.2	(1.3 - 3.7)	0.0039	0.0108	1.7	(1.1 - 2.7)	0.0183	0.0753	1.8	(1.1 - 2.8)	0.0161	0.0589	1.7	(1.1 - 2.6)	0.0296	0.2973
Multiple	3.4	(1.7 - 6.7)	0.0006	0.0023	1.6	(0.9 - 3.1)	0.1549	0.3591	2.3	(1.2 - 4.5)	0.0113	0.0589	1.2	(0.6 - 2.2)	0.6355	0.699
Complement	2.1	(1.2 - 3.9)	0.0185	0.0346	1.9	(1.1 - 3.3)	0.0205	0.0753	1.8	(1 - 3.2)	0.0392	0.1078	1.5	(0.9 - 2.6)	0.171	0.3751
Autoinflammation	1.7	(0.9 - 3.2)	0.1265	0.1989	1.5	(0.8 - 2.6)	0.1959	0.3591	1.3	(0.7 - 2.3)	0.3909	0.5312	1.6	(0.9 - 2.8)	0.1467	0.3751
Combined Syndromic	6.1	(2.5 - 14.8)	0.0001	0.0006	2.9	(1.2 - 7.2)	0.0195	0.0753	7.8	(2.2 - 27)	0.0002	0.0022	2.6	(1 - 6.6)	0.0541	0.2973
Innate	3.4	(1.3 - 9.2)	0.0189	0.0346	1.5	(0.6 - 3.9)	0.4721	0.6275	2.9	(1 - 8.5)	0.0542	0.1193	2.1	(0.8 - 5.9)	0.1558	0.3751
Phagocyte	1.5	(0.4 - 5.2)	0.5012	0.5513	0.6	(0.2 - 1.9)	0.5836	0.6419	2.7	(0.8 - 8.8)	0.11	0.2017	1.4	(0.5 - 4.1)	0.5989	0.699
Dysregulation	7	(2.3 - 21.1)	0.0005	0.0023	2.1	(0.7 - 6)	0.1949	0.3591	1.6	(0.6 - 4.7)	0.4346	0.5312	1.5	(0.5 - 4.4)	0.5968	0.699
Bone Marrow	1.3	(0.3 - 4.9)	0.719	0.719	2	(0.6 - 6.4)	0.2609	0.4099	1.1	(0.4 - 3.5)	1	1	1.1	(0.3 - 3.3)	1	1
Ab	2.8	(0.7 - 10.7)	0.2157	0.2637	0.5	(0.1 - 2.1)	0.5134	0.6275	2.3	(0.6 - 9.1)	0.3303	0.5191	0.4	(0.1 - 1.6)	0.2046	0.3751
Cellular & Humoral	4.2	(0.6 - 31.3)	0.1808	0.2485	1.2	(0.2 - 9.1)	1	1	1	(0.1 - 7.1)	1	1	0.3	(0 - 3)	0.3548	0.5575
